# Barriers to Institutional Childbirth in Rumbek North County, South Sudan: A Qualitative Study

**DOI:** 10.1371/journal.pone.0168083

**Published:** 2016-12-15

**Authors:** Calistus Wilunda, Chiara Scanagatta, Giovanni Putoto, Risa Takahashi, Francesca Montalbetti, Giulia Segafredo, Ana Pilar Betrán

**Affiliations:** 1 Department of Pharmaco Epidemiology, Graduate School of Medicine and Public Health, Kyoto University, Kyoto, Japan; 2 Doctors with Africa CUAMM, Padua, Italy; 3 Department of Nursing Science, Faculty of HealthCare, Tenri Health Care University, Nara, Japan; 4 Doctors with Africa CUAMM, Maper, South Sudan; 5 UNDP/UNFPA/UNICEF/WHO/World Bank Special Programme of Research, Development and Research Training in Human Reproduction, Department of Reproductive Health and Research, World Health Organization, Geneva, Switzerland; University of Bristol School of Social and Community Medicine, UNITED KINGDOM

## Abstract

**Background:**

South Sudan has one of the world’s poorest health indicators due to a fragile health system and a combination of socio-cultural, economic and political factors. This study was conducted to identify barriers to utilisation of institutional childbirth services in Rumbek North County.

**Methods:**

Data were collected through 14 focus group discussions with 169 women and 45 men, and 18 key informant interviews with community leaders, staff working in health facilities, traditional birth attendants, and the staff of the County Health Department. Data were analysed using inductive content analysis.

**Results:**

The barriers to institutional childbirth were categorised under four main themes: 1) Issues related to access and lack of resources: long distance to health facilities, lack of transportation means, referral problems, flooding and poor roads, and payments in health facilities; 2) Issues related to the socio-cultural context and conflict: insecurity, influence of the husband, lack of birth preparedness, domestic chores of women, influence of culture; 3) Perceptions about pregnancy and childbirth: perceived benefit of institutional childbirth, low childbirth risk perception, and medicalisation of childbirth including birth being perceived to be natural, undesirable birth practices, privacy concerns, and fear of caesarean section; and 4) Perceptions about the quality of care: inadequate health facility infrastructure and perceived neglect during admission.

**Conclusions:**

Multiple factors hinder institutional childbirth in Rumbek North. Some of the factors such as insecurity and poor roads are outside the scope of the health sector and will require a multi-sectoral approach if childbirth services are to be made accessible to women. Detailed recommendations to increase utilisation of childbirth services in the county have been suggested.

## Introduction

Reduction in maternal mortality is still a top global development agenda as reflected in the third Sustainable Development Goal [1]. Although some progress was achieved in reducing maternal mortality during the era of Millennium Development Goals, many countries, especially in sub-Saharan Africa, failed to achieve the targets of this goal [2]. Globally, the number of maternal deaths fell 44% between 1990 and 2015 [3]. In 2015, almost all global maternal deaths (99%) occurred in developing countries, and sub-Saharan Africa alone accounted for 66% of the deaths [3]. About 63% of maternal deaths occur intrapartum or postpartum [4]; thus, access to high quality skilled care around the time of childbirth can reduce maternal mortality.

A fragile health system and poor socioeconomic status exacerbated by decades of conflict have resulted into poor health indicators in South Sudan. For instance, a 2010 survey showed that the coverage of skilled attendant at childbirth was 19.4% and that of caesarean section was 0.6% [5]. Although a report by United Nations agencies and the World Bank has shown that maternal mortality ratio (MMR) declined in South Sudan by 54.4% between 1990 and 2015 [3], a different study has shown that MMR increased in the country from 763·8 per 100,000 births in 1990 to 956·8 per 100,000 births in 2013 (a 25.3% rise), and is projected to remain in the range of 500–925 per 100,000 livebirths by 2030 [4]. Access to health services in South Sudan is hampered by a poorly functioning health system that is plagued by chronic problems such as shortage of human resources, lack of health infrastructure and supplies, and weak management [6].

There is a dearth of studies on barriers to institutional childbirth in South Sudan. Some qualitative studies have reported that distance to health facilities, urgent deliveries, lack of transport means, lack of money to cover indirect costs, decision by husband on place of delivery and fear of a caesarean section were barriers to institutional childbirth in the country [7, 8]. A quantitative study has revealed that women’s education, utilisation of antenatal care (ANC), experience of complications during pregnancy, and living in urban areas were positively associated with use of skilled birth attendants [9].

The health system in South Sudan relies heavily on donor funded programs [10]. Based on the “contracting out” model [11], non-governmental organisations are the primary health services providers in the country [12]. In 2013, Doctors with Africa CUAMM (hereafter referred to as CUAMM) was chosen to partner with the Ministry of Health in Rumbek North County to implement a county-wide comprehensive primary health care project. With virtually no health care system in place, the project prioritised the reactivation of health facilities which had been closed or were partially functioning due to lack of staff, equipment and supplies. The reactivation involved renovation of health facilities; staff recruitment, on-the-job training and supervision; and provision of equipment, drugs and supplies. However, after about one year, almost all women were still delivering at home despite the availability of free-of-charge childbirth services in the county. This study was conducted to understand the barriers to utilisation of institutional childbirth services in the county; the findings of which were to inform the design of a strategy to redress the problem.

## Materials and Methods

This study is reported according to the consolidated criteria for reporting qualitative research (COREQ) [13], and the COREQ checklist is presented in S1 Table.

### Study area

This study was conducted in Rumbek North County which in 2015 had a population of 59,740 people, based on the 2008 census projection [14], and was divided into 6 payams (sub-county units): Alor, Malueth, Mayen, Madol, Maper and Wunrieng. The county’s population is semi-nomadic and pastoralism is the main economic activity. As the population moves, it establishes temporary settlements called “cattle camps”. In 2015, the county had one Primary Health Care Centre (PHCC) located in Maper, and seven Primary Health Care Units (PHCUs). Each one of the PHCUs was run by one community health worker (CHW), one traditional birth attendant (TBA), and one drug dispenser. The PHCC had three professional health workers: a nurse, a midwife and a clinical officer (a holder of a three-year diploma in medicine); all expatriates. The nearest hospital from Maper PHCC is Rumbek State Hospital; located 100 Km away. South Sudan has a decentralised health system that is based on four levels of administrative structures: central, state, county, and community [10]. PHCUs and PHCCs are, respectively, the lowest and second lowest health facilities situated at the community (sub-county) level. PHCCs are mandated by the Ministry of Health to provide childbirth services; including emergency obstetric care, while PHCUs, depending on the availability of qualified staff, are supposed to attend to normal (uncomplicated) deliveries [15]. Each PHCU is supposed to be staffed by two CHWs and a community midwife while a PHCC is supposed to have one clinical officer, three professional nurses, two midwives, three CHWs, and lower cadre staff [15]. Routine data show that, in 2014, out of the expected 3278 childbirths in the county, only 36 (1.1%) were assisted by a skilled birth attendant and 56 (1.7%) took place in any health facility.

### Design

This is a cross-sectional qualitative study that collected data utilising focus group discussions (FGDs) and key informant interviews (KIIs). FGDs were used to explore how different factors influence an individual’s decisions and perceptions related to care seeking for institutional childbirth. KIIs were used to gather in-depth information on the situation of maternal health care in the county and to triangulate some of the information gathered during FGDs.

### Sampling methods and participants

Villages in the country were stratified by payam and randomly selected as follows: two villages from each of Malueth and Mayen payams (the most populous) and one from each of Madol, Alor, Maper and Wunrieng. In each village, one FGD was conducted with women who had delivered in the past one year. Additionally, in a random sub-sample of half of the villages, husbands of women who delivered in the past one year were recruited to participate in men’s FGDs. Two extra FGDs with women were conducted in one cattle camp that was accessible during the study period. In each selected village, CHWs with the help of the village leaders invited (via face-to-face) 12 eligible participants to take part in the study. This was done one or two days before the respective FGD. The number of women who turned up for FGDs was often slightly higher than expected, and all were included.

KIIs were conducted with the following categories of individuals: 1) CHWs in the PHCC and PHCUs, 2) TBAs (those working in health facilities and those in the community), 3) community leaders, and 4) staff at the County Health Department (CHD). Participants of KIIs were purposively selected in consultation with the project staff and were individuals perceived to be knowledgeable about the maternal health situation in Rumbek North.

### Data collection

FGDs and KIIs were conducted in March 2015 utilising open-ended pretested question guides (S1 File). Each FGD was conducted by two Dinka (local language) speaking facilitators who were previously unknown to participants. The facilitators were of the same gender as participants, had at least high school level education, and were well versed with the local language and culture. One data collector was in charge of facilitating the sessions while the other one managed audio recordings and took field notes. The data collectors were trained for one day and were supervised by one of the co-authors (CW) who has experience in conducting qualitative studies and was present at all FGDs. The venues for FGDs were suggested by community leaders and often included local churches and under tree shades. KIIs took place at venues that were convenient to participants following prior arrangements with the study team.

**Fig 1:**
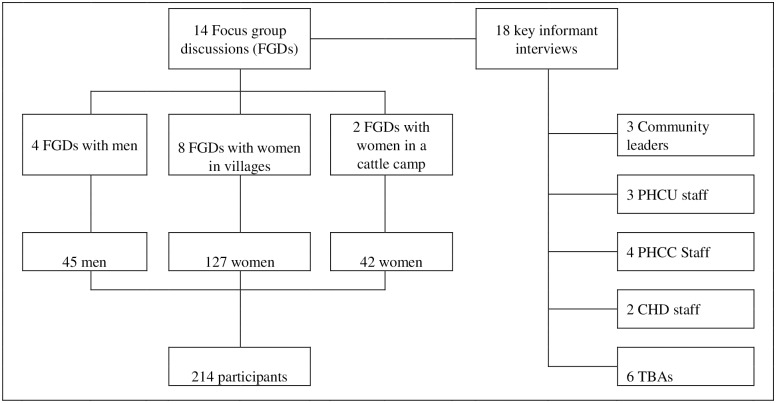
Participants’ flow chart. CHD: County Health Department; PHCC: Primary Health Care Centre; PHCU: Primary Health Care Unit; TBA: Traditional birth attendant.

Based on time and logistical constraints, a total of 14 FGDs and 18 KIIs were conducted (Fig 1). Women’s FGDs had a median of 16 participants while men’s FGDs had a median of 9 participants. To avoid interruptions and to maintain privacy, other people who approached the groups were kindly requested to leave. There were no drop-outs during FGDs. All KIIs were conducted by one of the co-authors (CW) either directly in English (for CHWs and CHD staff) or through a translator (for the other type of informants). Both KIIs and FGDs were audio recorded. Each FGD session lasted for about one hour whilst each KII lasted for about 20 minutes. No repeat interviews were conducted.

### Data analysis

The audio recordings in Dinka were transcribed and translated into English by bilingual (Dinka and English) speakers while the recordings of KIIs conducted in English were transcribed by CW. It wasn’t logistically possible to return the transcripts to participants for review. The transcripts were edited and read through severally to obtain an overall picture and to identify emerging patterns. CW analysed the transcripts based on the inductive content analysis approach [16]. The analytic framework was adapted from a published systematic review of qualitative studies on determinants of delivery care use in low- and middle-income countries [17]. The framework contains four main themes: 1) Access and resource availability, 2) Influence of the socio-cultural context, 3) Perceptions of pregnancy and delivery, and 4) Perceptions of the quality of care [17]. Each transcript was entered into NVivo 10 (QSR International, Melbourne, Australia) where a coding frame had been set up using the key themes (and sub-themes) from the analytic framework. Each transcript was then read through and segments of the text that captured the predefined theme were coded. New emerging themes were also identified and coded. This was done is a similar way for all FGDs. Information from KIIs was used to triangulate findings from FGDs. The data for each theme and sub-themes were then pieced together to provide an overview of the content relating to that specific theme or sub-themes. Quotes were selected to represent a typical response or to illustrate a deviant opinion. Participants did not provide feedback on the findings.

### Ethical considerations

This study was approved by the Ministry of Health Ethics Committee and by Rumbek North CHD. Because the participants of FGDs, TBAs and community leaders were either illiterate or of low literacy levels, they provided verbal informed consent, which was audio recorded, after an explanation about the study. The ethics committee had approved this method of obtaining consent. CHWs and staff of the CHD provided written consent. Permission to conduct the study in selected villages was sought from village leaders. All collected information including audio recordings and transcripts were securely kept and were accessible only to the research team. Each participant of FGDs received a bar of soap to compensate for his/her time. No monetary incentives were provided.

## Results

The median age of female FGD participants was 25 years, about 97% had no education, and 92.6% were currently married (Table 1). Most of the participants (67.5%) attended some ANC during their most recent pregnancy but only a minority (5.9%) delivered in a health facility. The median age of male FGD participants was 35 years and most of them (71%) had no formal education.

**Table 1:** Characteristics of female focus group discussions participants.

Characteristic	Frequency (n = 169)	Percent
**Age group**		
<20	21	12.4
20–24	52	30.8
25–29	58	34.3
>29	38	22.5
Median (IQR)	25 (20–29)	
**Parity**		
1	27	16.0
2–3	55	32.5
4–5	64	37.9
>5	23	13.6
**Education level**		
None	163	96.4
At least primary	6	3.6
**Marital status**		
Currently married	156	92.3
Formerly married	13	7.7
**Attended ANC last pregnancy**		
Yes	114	67.5
No	55	32.5
**Place of delivery for last pregnancy**		
Home	159	94.1
Health Facility	10	5.9

The perceived barriers to institutional childbirth are summarised in Table 2 and presented below in detail based on the themes in the analytic framework. Participants often used the word hospital to mean any type of health facility.

**Table 2:** A summary of barriers to institutional childbirth in Rumbek North County.

Barrier	Key findings
**1. Access and resource availability**	
1.1. Transportation/access	
1.1.1. Proximity of health facility	Long distance to heath facilities. Semi-nomadic lifestyle in search for water and pasture increased the distance to health facilities.
1.1.2. Transport means availability	Lack of commercial or private means of transportation to health facilities during labour.
1.1.3. Floods and poor roads	Floods and mud during the wet season, parts of roads being washed away by floods, inaccessibility of health facilities for delivery of drugs and supplies, break down of the referral system because the ambulance cannot move
1.1.4. Referrals	Poorly functioning referral system due to: lack of communication means at PHCUs; lack of transportation means and long distance to the PHCC; and floods and poor roads during wet seasons.
1.2. Costs	Women are charged at some health facilities. Payment during the past institutional childbirth affected current use. TBAs are affordable and are paid in kind.
**2. Influence of the socio-cultural context and conflict**	
2.1. Insecurity	Frequent attacks and fear of being attacked any time by neighbouring tribe/clans. Due to insecurity, women cannot leave children at home alone to go to a health facility and husbands do not allow their wives to deliver in a health facility. Displacement after attacks exacerbating geographic inaccessibility.
2.2. Influence of husband/ male partner	Husbands decide on place of delivery. Men restrict their wives from delivering in a health facility. Institutional delivery allowed only in case of complications. Fear of domestic violence for disobeying the husband.
2.3. Preparedness for childbirth	Lack of birth preparedness. Labour comes abruptly. Expected day of delivery unknown. Birth preparedness perceived to be unnecessary because of the uncertainty of the birth outcome. Husbands unsupportive of birth preparedness.
2.4. Women’s domestic chores	Preoccupation with domestic chores including taking care of children, taking care of the house and producing and preparing food for the family. Women left at home by men who are in the military or cattle camps. Lack of someone to leave behind with children if a woman decides to go to the heath facility.
2.5. Influence of tradition and culture	Cultural beliefs related to placenta handling. Throwing of the placenta in the pit is culturally unacceptable and is believed to cause infertility. Men insisting on women to deliver at home in order to rule out infidelity. Childbirth at home is a normal traditional practice. Beliefs around food consumption and shower after delivery.
**3. Perceptions of pregnancy and childbirth**	
3.1. Benefits of institutional childbirth unknown	Institutional childbirth perceived to be a new concept and hence lack of information about its significance. Ambivalent perceptions towards institutional childbirth. Lack of prior experience with institutional childbirth.
3.2. Low risk perception	Child birth perceived to be something simple and of low risk. This was influenced by tradition: foremothers used to deliver at home without any problem thus visiting a health facility was unnecessary.
3.3. Medicalization of childbirth	
3.3.1. Birth is a natural event	Childbirth perceived to be a natural event and hence did not require medical intervention. Institutional childbirth was necessary only in case of complications.
3.3.2. Supportive familiar companionship at birth	Home delivery is comfortable because of family members’ support. Family members/neighbours provide physical support when a woman is delivering in the squatting position; this kind of support is absent in health facilities.
3.3.3. Undesirable birth practice and privacy	Undesirable or unfamiliar birth practices during health facility delivery including: birthing position, having to remove all clothes, and vaginal examinations. Limited space at the health facility. Assistance by male heath workers.
3.3.4. Fear of caesarean section	Fear of caesarean section delivery in health facilities
**4. Perceptions of quality of care**	
4.1. Health facility infrastructure and commodities	Lack of physical infrastructure for maternity at health facilities. Specifically, lack of a separate maternity area; laboratory services; drugs; equipment; and qualified staff.
4.2. Neglect and lack of communication	Perception that health care workers neglect patients’ needs. Insufficient communication between health care workers and women.

### Access and resource availability

#### Transportation/access

Long distance to health facilities emerged across all FGDs and KIIs as a key barrier to institutional childbirth. At the time of this study, although TBAs working in PHCUs were attending to uncomplicated deliveries, in the entire county, only the PHCC had the staffing capacity required to offer childbirth services. The problem of distance was exacerbated by poor roads, lack of transportation means and a sparse population. Thus, childbirth services were geographically inaccessible to most of the population, as captured in the quote below:

“Meen hospital [PHCU] and Maper hospital [PHCC] are very far from us. We are actually in the middle between Rumkek and Maper hospitals. If you want to go to hospital, you can spend one day to reach there.”

(Female FGD participant, Wundhiot village)

Long distance to health facilities instilled in women a sense of powerlessness and resignation regarding the outcome of labour as illustrated below:

“Sometimes you might be in labour for three days, if Gods helps, you deliver safely, if not, you or the child might die because the hospital is very far.”

(Female FGD participant, Madhol village)

For women in cattle camps, the problem of distance to health facilities was aggravated by population movements, which sometimes increased the distance to health facilities, as highlighted in this statement:

“If you are in labour and are about to give birth, you cannot manage to walk for a long distance because the hospitals are very far. We people in the cattle camp can sometimes move very far in search of good grazing grounds. After some months, we might feel that the place where we are is not good for our cattle and so we may move to new a place.”

(Female FGD participant, Nhomleng cattle camp)

Women in labour could not reach health facilities because of lack of transportation means. This problem was aggravated by long distance to the health facilities and poor rugged roads as illustrated below:

“The hospital is very far from us and there are no means of transportation to take us there during labour.”

(Female FGD participant, Achiek village)

“…some [women] stay in the cattle camp and some stay very far, and without transportation means they cannot reach the health facility. You know this place doesn’t have means of transportation, so even if you have money you cannot facilitate yourself.”

(KII, CHD staff)

Although a free-of-charge ambulance service was available, it was facing several constraints which affected its operation, as explained below.

Another issue that emerged was floods and poor roads. Most parts of Rumbek North were prone to floods during the rainy season (usually from April to November). The floods swept away parts of the main road from Rumbek town, and other roads in the county. Consequently, most villages, including those near the PHCC, were completely cut off during rainy seasons. Additionally, the floods prevented the movement of the ambulance; paralysing the referral system. This problem is illustrated in this quote:

“I had decided to deliver in the hospital but it started raining and the road became flooded.”

(Female FGD participant, Meen village)

Poor roads and floods meant that in case of obstetric emergencies, pregnant women could only reach health facilities if they were carried by men as depicted below:

“This month of March is good, when it comes to August, there will be no way a car can come here because of floods. During that time, we put our ladies on our shoulders and take them to the hospital.”

(KII, Community leader, Malueth Payam)

Inaccessibility of health facilities due to floods sometimes resulted into fatal maternal and/or foetal outcomes as captured in this account of a TBA:

“….during the last rainy season, five women died because there was no way the vehicle could go and pick them. One of the women bled a lot after delivery and died. When people reached there with a motorbike, they found her already dead and they just brought the child whom we referred to Rumbek. Some children also die with their mothers. The women died in Meen Payam. There is another village called Obuoth, where a young girl who had been married at a pride price of 100 cows died during the delivery of her first child.”

(KII, TBA, Maper PHCC)

Lack of communication means at PHCUs, poor roads, lack of transportation means especially during the rainy season, and long distance to the PHCC affected the referral system. During wet seasons, the ambulance became unusable because of muddy roads and floods. In most cases, women with complications had to be carried to Maper PHCC by community members. Sometimes, during the dry season, staff at PHCUs had to trek for long distances to the PHCC to call for the ambulance. This problem is illustrated below:

“I tell the mother go to Maper. If she can walk and get there, she can go, if not, what can I do? I never call the ambulance because we do not have any means of communication. That radio is for the Payam Administrator and there is nobody who sits there. For a woman who is not able to walk, I tell the community members that if they are able, they take her on their shoulders up to the health facility in Maper. If there is nobody to take her, I send somebody or I even go by myself on foot to Maper PHCC to ask for the ambulance.”

(KII, CHW Alor PHCU)

#### Costs

FGD participants mentioned that women were being charged for childbirth services at some health facilities, and this deterred institutional childbirth. It wasn’t clear whether these were official or “under-the-table” payments as participants could not tell the difference. It also emerged that women were mostly being charged at health facilities outside the county, particularly at the hospital in Rumbek. The past experience of having been charged user fees at any health facility seemed to instil a negative attitude towards future use of institutional childbirth. These sentiments are highlighted in the following quotes:

“What I dislike about the hospital is that after delivery, the mother is asked to pay money, but we don’t have money; we just go there to get help.”

(Female FGD participant, Meen village)

“When I was pregnant, I decided to deliver in the hospital but when I went to the main hospital in Rumbek, I was asked to pay money in the maternity. By then this hospital [PHCC] in Maper had not yet been opened and so I came back to deliver at home.”

(Female FGD participant, Maper centre)

On the other hand, delivering at home assisted by a TBA was rarely associated with direct financial costs. Women were free to give whatever material item they could afford, as illustrated below:

“The woman who has been assisted can give a calabash bowl, tobacco or alcohol to the TBA.”

(Female FGD participant, Achiek village)

“In our tradition, after helping a pregnant mother during delivery, she [the mother] has to pay something like a calabash bowl, a basin or soap. In the past you could pay tobacco or alcohol.”

(KII, TBA, Malueth PHCU)

### Influence of the socio-cultural context and conflict

#### Insecurity

Irrespective of the prevailing security situation in South Sudan, frequent attacks from neighbouring communities and inter-clan feuds meant that Rumbek North was in a state of chronic insecurity and this was negatively effecting the general utilisation of health services. The attacks often led to displacement of people and destruction of property including health facilities. Women could not leave their children at home unattended and go to deliver in a health facility because of the fear of an attack as illustrated below:

“Our place is also in the middle of enemies who frequently attack us. Some of us fear that if we go to deliver in the hospital and the enemy comes to attack in our absence, there will be nobody to lead our children to a hiding place.”

(Female FGD participant, Achiek village)

#### Influence of husband/male partner

Male partners wielded inordinate influence on the place of childbirth. It was consistently mentioned that the final decision on the place of childbirth rested with the husband, and in the absence of an overt obstetric complication, many men were restricting their partners from delivering in health facilities as highlighted below:

“The man is the one who married her and he decides where she will give birth.”

(Male FGD participant, Achiek village)

“…our men are the ones who refuse to allow us to go to the hospital. Everybody says that the hospital is good but you know women stay under men’s control. Even if you tell him what you want, if he doesn’t agree with you, he will not allow you to go to the hospital.”

(Female FGD participant, Madhol village)

Men were restricting women from institutional childbirth because: 1) leaving children and homes unattended was unacceptable, 2) childbirth was perceived to be an easy natural process that did not warrant going to a health facility, 3) the presence of male birth attendants in health facilities, and 4) power relations in the family: men had the overall authority on childbirth place. This is captured in the following statements:

“The husband is the one who decides where a woman should give birth. Even if a woman has decided to deliver in the hospital the husband will say ‘no, you are just going to roam there, you must deliver here. Whom will you leave your children with if you decide to go and deliver in the hospital?’ Our husbands decide where we should deliver.”

(Female FGD participant, Meen village)

“During my last pregnancy, I told my husband that I need to deliver in a health facility but he refused to allow me; saying that he did not want me to be seen by men while I am delivering. I delivered at home to twins and both of them died. I remained regretting for not having delivered in the hospital.”

(Female FGD participant, Maper centre)

Women who took it upon themselves to deliver in a health facility were considered rebellious and risked being subjected to domestic violence.

#### Preparedness for childbirth

Pregnant women were not making any preparations for childbirth. For most women, labour came abruptly with no time to go to a health facility. This perception is partly an indication of lack of preparedness. It also emerged that women did not know the expected date of delivery and thus could not prepare for child birth as captured in the following quotes:

“We don’t go to the hospital because we don’t know the day when the baby will come out. If we knew the day of delivery, we would be preparing ourselves.”

(Female FGD member, Wundhiot village)

“Traditionally, we prepare nothing. We stay like that without preparing anything until delivery. The baby is delivered like that on the floor or under a tree.”

(Female FGD member, Nhomleng cattle camp)

In some cases, birth preparedness was seen as unnecessary and a potential source of shame because the birth outcome was uncertain as portrayed in this statement:

“We don’t prepare anything before delivery. This is because some babies die before spending a day. Now imagine if you had prepared inside your house, you will regret for having bought those things for nothing because your baby will not be there. For this reason, some women like me don’t like such a shame. The only thing you do is to ask God to help you during delivery.”

(Female FGD participant, Maper centre)

Husbands were also against any birth preparations as depicted below:

“Last time when I was pregnant, I asked my husband to buy me pieces of cloth, soap, sugar, cups, bed sheets and a baby carrier and he said, ‘why should I buy for you such items before you give birth? ……those things will be bought after you deliver. Where on earth can clothes of a baby be bought while the baby is still in the womb? Who knows whether the baby will come out alive or dead?’”

(Female FGD participant, Maper centre)

#### Women’s domestic chores

Women in Rumbek North bear a heavy burden of domestic chores: they are responsible for taking care of children, taking care of the house, and producing and preparing food for the family. Women whose husbands were working away from home, for instance in the military or in cattle camps, seemed to be greatly disadvantaged. Having to take care of young children seemed to bear the highest weight on preventing women from delivering in health facilities. The problem of domestic chores was compounded by the long distance to health facilities and insecurity. Women who desired to deliver in a heath facility often lacked someone to take care of their children left at home. The following quotes illustrate these issues:

“For my case, I am the only woman at home and there is nobody else to help me. Therefore, my husband cannot allow me to deliver in a health facility because there will be nobody to cook for children, and all domestic work at home will remain undone.”

(Female FGD participant, Maper centre)

“Sometimes, our husbands would say that they need to take cattle to a place where there is enough pasture and water. We are then left behind to take care of the home, with nobody else to leave children with in case we want to go to the hospital.”

(Female FGD participant, Wundhiot village)

#### Influence of tradition and culture

Tradition and culture influenced the place of childbirth. Women preferred to deliver at home and assisted by someone from the same area partly to ensure that the placenta was properly handled according to the culture as highlighted below:

“The other reason why delivering with the help of TBAs is good is that the placenta is handled properly; it is washed, put in the tin and covered. A hole is then dug and it is buried. This is what I like about delivering at home.”

(Female FGD participant, Biar village)

A man would insist on his wife delivering at home if he suspected that the baby being expected was not his. It was believed that if the child belonged to another man, the woman would not deliver until she mentions the name of the man. Thus, men viewed home delivery as an opportunity to prove the fidelity of their wives as highlighted below:

“If the husband suspects that his wife committed adultery, he will insist on her delivering at home so that she can mention the name of the man with whom she committed adultery. If the woman committed adultery, the baby will not come out until she mentions the name of the father. Usually the midwife would hide this information from the husband, and because of this, men do not allow their wives to deliver in the health facility.”

(Male FGD participant, Meen village)

For some women, delivering at home was conventional because it was the way of life as passed down through generations, and there was no need for change because it had worked well in the past as illustrated below:

“Long time ago, our great grandmothers never delivered in the hospital, but delivered at home. For this reason, we prefer to deliver at home”

(Female FGD participant, Biar village)

### Perceptions of pregnancy and delivery

#### Benefits of institutional childbirth unknown

It was generally perceived that delivering in the health facility was a new concept in Rumbek North, and its advantages over home delivery were unknown. This perception seemed to bear higher weight on the populations that lived far away from health facilities or in cattle camps as illustrated blow:

“We have never given birth in the hospital but we deliver here in the cattle camp. We therefore don’t know the goodness or badness of delivering in the hospital.”

(Female FGD member, Nhomleng cattle camp)

“We never deliver in health facilities because we don’t know the advantage of delivering there. We just deliver at home.”

(Female FGD member, Chatom village)

Although women were ambivalent about the benefits of institutional childbirth from a maternal/neonatal health perspective, they were quick to mention the advantages of institutional childbirth from a material/incentive perspective. The baby kits that women delivering at the PHCC were receiving were widely appreciated and this was starting to encourage institutional births as depicted below:

“If you go to deliver in the hospital, the midwife will prepare everything for your delivery such as pieces of clothes and soap for washing the baby, a cup, and a basin for bathing.”

(Female FGD member, Biar Village)

“The number of women delivering in the health facility could not reach five [per month], but now they are starting to come. They are encouraged to come because of the incentives and the free-of-charge delivery.”

(KII, CHW Maper PHCC)

#### Low risk perception

Childbirth was perceived by some to be simple and with low risk. This perception was often influenced by tradition and culture as described above. Both men and women felt that because their grandparents used to deliver at home without any problem, there was no need of going to the health facility for childbirth as captured in these statements:

“We believe that childbirth is one of the simplest things one can do because even our great grandmothers did it without any problem.”

(Male FGD participant, Achiek village)

“Our grandmothers never went to hospitals but they gave birth normally and so we follow the way they used to give birth.”

(Female FGD participant, Meen Village)

#### Medicalization of childbirth

Childbirth was perceived to be a natural process that did not require visiting a health facility if there was no complication. Additionally, labour pain was considered to be something trivial. The implication of these perceptions was delayed decision making in seeking health care as illustrated below:

“It is until you are in labour for almost two days that you will be taken to the hospital to deliver there. The reason you go to the hospital is because of severe labour pain. With the little pain you experience and then give birth, do you think you need to go to the hospital?”

(Female FGD participant, Biar village)

“A man can decide where his wife should give birth after realising that she is having difficulties in delivering.”

(Male FGD participant, Maper centre)

Women were comfortable with home childbirth because of the support they got from family members and/or neighbours around the time of childbirth. Family members or neighbours could not travel to the health facility to support a woman partly because of the long distance. Women giving birth required physical and emotional support but such support was often unavailable at health facilities as illustrated below:

“Giving birth at home or in the cattle camp is not bad because there will be many people to help you. For instance, some people will come and hold your shoulders and another one will put you on her laps. If it is in the hospital, there is only the midwife to help you.”

(Female FGD participant, Nhomleng cattle camp)

“They are also concerned about lack of somebody to take care of them when they come to deliver here. Also, this facility has no food to eat.”

(KII, CHW Maper PHCC)

Childbirth position and vaginal examination emerged as two key birth practices that women disliked in health facilities. Women preferred the squatting position partly because it ensured minimum exposure. On the contrary, they disliked the lithotomy position and the removal of clothes, as is done in health facilities. These issues are articulated in the following quotes:

“We don’t like the delivery position in the hospital whereby a woman lays down and opens her legs wide. It is not a good position of giving birth according to our imagination. But in the village or at home, we just squat with legs slightly open and somebody pushes the mother down on the shoulders while the TBA is in front waiting for the baby.”

(Female FGD participant, Meen village)

“Women don’t like vaginal examination. In the hospital, they put on gloves to do vaginal examination to measure whether the woman will deliver well or not, and women do not like this.”

(KII, TBA, Maper PHCC)

There was also the issue of privacy that was related to limited maternity infrastructure resulting into women sharing facilities with general inpatients, and birth attendance by male health workers as highlighted in the following quotes:

“The clinic is very small and it cannot accommodate many people. Some people don’t want to come because the health facility is too crowded with many people mixed up. They say that they don’t want to be seen by many people.”

(KII, CHW Maper PHCC)

“…in the hospital, men are the ones who help you during delivery, and we don’t like men to see us naked. I don’t like that kind of delivery. Even if there are so many doctors to help us in the hospital, I will never go.”

(Female FGD participant, Madhol village)

Some women preferred to give birth at home because they feared caesarean delivery as highlighted below:

“…..they are afraid of caesarean section. They are worried about not being able to continue producing children after caesarean section.”

(Female FGD participant, Madhol village)

However, this perception was not universal as some women and men felt that caesarean delivery, in case of an obstetric complication, was one of the advantages of delivering in the health facility as captured in the quote below:

“The hospital is very good because if your baby cannot come out quickly, the doctors will decide whether to operate on you or not. If the baby has died, they can remove it from your womb and you will remain healthy.”

(Female FGD participant, Biar Village)

“The advantage of delivering in a health facility is that if a woman is having difficulty with birth, the doctors can operate on her to remove the baby.”

(Male FGD participant, Meen village)

### Perceptions of quality of care

#### Lack of infrastructure and commodities

Participants, especially health workers, lamented of lack of physical infrastructure to support provision of childbirth services at all health facilities. Although Maper PHCC had a maternity unit, postpartum women were forced to share the only available ward with general inpatients. Participants also complained of lack of drugs, equipment, staff, and laboratory services at health facilities especially at PHCUs. Overall, participants had a low perception of the quality of care provided at PHCUs. They perceived that the “good” drugs (injectable) available at the PHCC were unavailable at the PHCUs. This issue is highlighted in the following statements:

“…..we lack a good room where a woman can deliver. What is here in Malueth? The main medicines for certain diseases are not there. Those medicines are there in Maper. Even if a pregnant woman is about to deliver, we don’t have a good room where she can deliver. We have to take that woman to the hospital.”

(KII, Community leader, Malueth Payam)

“This maternity is not enough. Now all patients including women are being admitted in one ward.”

(KII, CHW Maper PHCC)

“In the health facility, one midwife is not enough to attend to delivering mothers at the same time. Other staffs are not well trained.”

(Female FGD participant, Achiek)

#### Neglect and lack of communication in health facilities

Related to lack of supportive care during childbirth in health facilities, some women who had previously delivered in a health facility felt that no attention was paid to their basic needs. They were also not informed in advance about what would not be provided in health facilities during admission to allow them to make their own arrangements. This problem is captured in the quote below:

“….I was just put in a car to take me to the hospital and after I had delivered, the midwife and the doctors left me alone in the maternity and went away. I slept there without water for showering and food. That was not good. When somebody is not there to take care of you, it is better to deliver at home.”

(Female FGD participant, Meen village)

## Discussion

This qualitative study investigated perceived barriers to institutional childbirth in Rumbek North, South Sudan. Overall, our findings compare well with those of a recent systematic review that compiled information from Africa, Asia, South America and Middle East [17].

### Access and lack of financial resources

Availability of financial resources and accessibility to health facilities are key determinants of maternal health service use [17, 18]. Issues such as long distance to health facilities, lack of transportation means and lack of money to pay for transportation have also been reported as barriers to institutional childbirth in another study in South Sudan [7]. Rumbek North is a marginalised area with extremely poor infrastructure, and the few available roads are in a dire condition and cannot attract private investment in transportation. Most villages are connected to PHCUs only by footpaths. The flat terrain coupled with poor drainage of water predisposes the land to perennial floods which last several months [19]. Reaching any kind of health facility was a challenge and this had a negative effect on the demand and supply of health services. Furthermore, although PHCUs were attending to normal deliveries, only the PHCC had one professional midwife, thus, access to a skilled birth attendant for a majority of the population remains a mirage.

In Rumbek North, sparse population settlements and population movements contribute to long distance to health facilities. There is also a long distance to referral health facilities, such as Rumbek Hospital and Marial Lou Hospital, located in the neighbouring counties. In some of these external health facilities, staff might have been requesting for “under-the-table” payments, even though maternal health services are supposed to be offered free-of-charge. This is in line with the findings that “under-the-table” payments were hindering institutional childbirth in South Sudan [7]. Long distances to health facilities and direct and indirect costs were exacerbating the impact of socio-cultural dynamics on women’s access to and utilisation of health services, as also explained below.

### Influence of the socio-cultural context and conflict

The socio-cultural context of Rumbek North had a negative influence on institutional childbirth. Domestic chores imposed on women by the society on the basis of tradition and culture, power relations which favour men, and women’s lack of autonomy in decision making on maternal health service use are formidable socio-cultural contextual barriers to maternal health service use in the county. The finding that husbands restrict their wives from utilising institutional childbirth services has also been reported in South Sudan [7]. Men perceived giving birth to be a women’s natural duty that had always been performed without specific arrangements. A study in Bangladesh found that, husbands whose wives utilised TBAs at home were uninvolved during childbirth and believed childbirth should take place at home according to local traditions, while those who delivered in a health facility had more supportive husbands [20]. A systematic review has also reported similar findings [17]; underscoring the need for interventions targeting male partners/husbands if utilisation of childbirth services is to be increased.

Domestic chores that women are expected to perform has been found to be a barrier to institutional childbirth in a similar context in Uganda [21]. Women delivered at home because there was nobody at home to leave their children with if they chose to deliver in a health facility. In addition, Rumbek North continues to be in a state of chronic insecurity, which negatively affects both health services provision and use. Indeed, frequent fighting in South Sudan is restricting access to health facilities through displacement of populations and destruction of health facilities [22]. Women in Rumbek North were on high alert for fear of being attacked by neighbouring communities at any time. Consequently, they couldn’t risk leaving their children at home and go to the health facility.

### Perceptions of pregnancy and childbirth

The role of tradition and culture in influencing institutional childbirth in different contexts is well documented [21, 23], and the present study confirms this. Families were not making any plans such as saving money, identifying means of transportation or moving close to a health facility, in preparation for childbirth. For some families, it was a taboo to plan for childbirth because of the uncertainty of the pregnancy outcome. The intention to deliver in a health facility was lacking, yet this is a key determinant of planned behaviour change [24]. Giving birth was perceived to be a routine event and a duty that every woman should perform, without prior arrangements, as foremothers did. Consequently, institutional childbirth was considered only if complications arose. This observation has also been reported elsewhere [9, 12].

Issues such as availability of familiar support during home delivery, undesirable birth practices, and lack of privacy in health facilities have also been documented as barriers to institutional childbirth in other studies [21, 25]. In line with the findings of this study, studies conducted elsewhere [17] and in South Sudan [7], have cited fear of caesarean section as a barrier of institutional childbirth. In Rumbek North, this appears to be fuelled by the perception that the procedure imposes a limitation on the number of children a woman can give birth to rather than the feeling that it is an unnatural intervention as has been reported elsewhere [26, 27]. Some women also thought that it was impossible to conceive again after caesarean delivery. However, not all participants had a negative perception towards caesarean delivery. In the context of the increase in caesarean section rates worldwide including in low-resource settings and countries [28], the situation in Rumbek depicts the global inequities in the use of this life saving procedure.

Perceived benefit is a key determinant of institutional childbirth [18]. Lack of correct and sufficient information about institutional childbirth, social norms related to pregnancy and childbirth and lack of prior use of formal health care could be the underlying reasons for poor perceived benefit of institutional childbirth and the low individual risk perception regarding home childbirth. Most women in Rumbek North, especially those in cattle camps, had no prior experience of institutional childbirth and were thus ambivalent about its usefulness. Lack of knowledge about maternal health has also been identified as a key barrier to institutional childbirth in Kenya [29].

### Perceptions about quality of care

Perceptions about the quality institutional childbirth were related mainly to poor facility infrastructure, perceived neglect, and lack of communication. Perceived quality of care is a known determinant of maternal service use and delays the decision to seek care [30], however, as most women had never delivered in a health facility, perceived quality of care did not emerge as strong barrier to institutional childbirth in this setting.

### Strengths and limitations

This is the first study on barriers to institutional childbirth in Rumbek North, and is also one of the few studies of its kind in South Sudan. The study had a large sample size and used two complimentary methods to collect data from diverse sources. This together with the triangulation of the data ensured that the main perceptions prevalent in the community were captured. However, the study has some limitations. It is possible that some information was lost during translation and transcription. Despite the training provided, FGD facilitators were inexperienced in qualitative research. Nevertheless, the quality of their work improved along the way as they gained experience and received feedback from the principal investigator.

### Conclusions and recommendations

Multiple factors affect the utilisation of institutional childbirth services in Rumbek North County. Some of the factors, such as insecurity and poor geographical inaccessibility, are outside the scope of the health sector and can only be tackled through a multi-sectoral approach in line with the principles of primary health care [31]. Most of the findings of this study are generalizable to other counties or settings with similar socio-cultural and economic profile as Rumbek North. Nevertheless, studies on this topic in other parts of the country are needed.

The scope of the challenges of institutional childbirth in this county is enormous and huge financial, human resources, insecurity, and logistical constraints still exist. Although the Ministry of Health no longer considers TBAs to be part of the formal health system, evidence has shown that TBAs can reduce perinatal deaths if well trained and supervised [32]. In the present context of Rumbek North, it is unrealistic to completely ignore TBAs. As has already been tried elsewhere [33], their role can be changed to that of promoters of institutional childbirth through sensitisation and referral of women to health facilities for ANC and childbirth services. Given that Rumbek North is a challenging context, continuous training and supervision of TBAs will be essential to ensure that they can conduct clean deliveries at home when it is not possible to refer, and that they can identify and promptly refer complicated cases. Institutional childbirth should still be encouraged. In health facilities, adopting the women’s preferred childbirth position as well as allowing the accompanying caretakers to be involved in the childbirth process should be considered. The problem of under-the-table payments should be investigated further and an appropriate action should be taken. Efforts should be made to provide food to women and their companions during the admission period. Awareness campaigns to promote safe motherhood targeting both men and women and involving community leaders should be pursued. There is also need to improve the health facility infrastructure and to lobby with county, state and national authorities to address the issue of poor roads and insecurity. Towards the end of this study, PHCUs were provided with motorcycles and radio communication equipment to facilitate referral of women and other patients.

## Supporting Information

S1 File Question guides for focus group discussions and key informant interviews. (DOCX)

S2 File A data file on barriers to institutional childbirth in Rumbek North. (PDF)

S1 Table Consolidated criteria for reporting qualitative studies: 32-item checklist. (DOCX)
