# Supplementary material for: Barriers to Institutional Childbirth in Rumbek North County, South Sudan: A Qualitative Study
Source: PLoS One. 2016 Dec 15;11(12):e0168083. doi: 10.1371/journal.pone.0168083 (PMC5158020; doi:10.1371/journal.pone.0168083)
Supplement: S1 File — (DOCX) [file pone.0168083.s001.docx]

**A. Women’s FGD question guide**

**Payam_______________Village: ____________Number of attendees: ______Date: ____________**

1. What are the roles of men in this community? What are the roles of women in this community?
2. Are there any health facilities in this county where a pregnant woman can be assisted during delivery?
3. What is your opinion about delivering in a health facility? Are there any disadvantages? What are they? Are there any advantages? What are they?
4. (For women who delivered at home): what are the reasons why who decided to deliver at home?
5. For women who delivered at in a health facility: what are the reasons why who decided to deliver in a health facility? Tell us about your experience when you delivered in a health facility
6. In general, what do you think are the reasons why some women in this village don’t deliver in health facilities (rephrase: what are some of the reasons why some women in this village deliver at home?) Probe further about traditions and beliefs around child birth
7. In this community, who decides where a woman should give birth?
8. What is your opinion about the care provided by traditional birth attendants during delivery?
9. Are there any preparations that a pregnant woman in this community has to make before the baby is born? What are these preparations?

**B. Men’s FGD question guide**

**Payam_____________Village: ________________Number of attendees: ______Date: __________**

1. What are the roles of men in this community? And are the roles of women in this community?
2. What is your opinion about delivering in a health facility? Are there any disadvantages? What are they? Are there any advantages? What are they?
3. In general, what do you think are the reasons why some women in this village don’t deliver in health facilities (rephrase: what are some of the reasons why some women in this village deliver at home?) Probe further about traditions and beliefs around child birth
4. In this community, who decides where a woman should give birth?

**C. Key informants’ interview guide (health staff)**

**Age: ______Sex: _______Cadre: ______________Payam: __________________Date: _____________**

1. For how long have you been working at this facility?
2. On average, how many deliveries do you attend to in a month
3. What is the longest distance (in km) do mothers have to cover to get to this facility?
4. What do you think about the quality care mothers receive at this facility?
5. Are there any areas for improvement?
6. Have you ever received any comments (negative or positive) from mothers regarding provision of maternal services at this facility? If yes, please tell us more about these comments?
7. In this community, what do you think are the main reasons why most women don’t deliver in health facilities?
8. What interventions can be introduced to encourage women to deliver at health facilities? (*interventions at both the community and the health facility*)
9. Are women sometimes asked to contribute something when they come to deliver at this health facility? Why do you think this happens?
10. For PHCU staff: Are women referred from this PHCU to the PHCC? Do you think the referral system of pregnant women from this PHCU to the PHCCs is working well? What are the challenges faced during referral. How can these be solved?

**D. Key informants’ interview guide (TBA)**

**Payam: _____________Date: ______Works in a health facility:** Yes/No **Name of HF____________**

1. For how long have you been working as a TBA?
2. On average, how many deliveries do you attend to in a month
3. Do you think women are satisfied with the type of maternity care they receive from TBAs? Why do you think so?
4. Have you ever received any comments (negative or positive) from mothers regarding provision of delivery care services by TBAs? If yes, please tell us more about these comments?
5. Where do you think pregnant women prefer to deliver?
6. Do you think it is necessary to encourage pregnant women to deliver in a health facility?

If yes, what can be done to encourage women to deliver in health facilities?

1. In this community, what do you think are the main reasons why most women don’t deliver in health facilities?
2. Do TBAs sometimes ask women to pay them something when they help them to deliver? What are they usually required/asked to pay?

**E. Key informants’ interview guide (Staff of County Health Department/local leader)**

**Age: _______Sex: ________Profession: _______________________Rank/position: ________________**

**Payam: ________________________Date:________________**

1. What is your opinion about provision of maternity care services in health facilities in this county? What do you think are the areas that need to be improved?
2. Have you ever received any comments (either positive or negative) from mothers regarding provision of maternal services at health facilities in this county? What are these comments?
3. Is there any strategy in place to encourage pregnant women to deliver at health facilities?
4. What role do you think TBAs are playing in delivery of maternal health services in this community?
5. In this county/community, what do you think are the main reasons why most women don’t deliver in health facilities?
6. What interventions can be introduced to encourage women to deliver at health facilities? (*interventions at both the community and the health facility*)
7. What are the main challenges of providing childbirth services to this county/community?
