# Supplementary material for: Barriers to Institutional Childbirth in Rumbek North County, South Sudan: A Qualitative Study
Source: PLoS One. 2016 Dec 15;11(12):e0168083. doi: 10.1371/journal.pone.0168083 (PMC5158020; doi:10.1371/journal.pone.0168083)
Supplement: S2 File — (PDF) [file pone.0168083.s002.pdf]

A data file on barriers to utilization of childbirth services in Rumbek North

Transportation/access

Proximity of health facility

<Internals¥¥FGD¥¥Men FGD¥¥Achiek_men 17032015_rev> - § 1 reference coded [3.12% Coverage]

Reference 1 - 3.12% Coverage

The other reason why they deliver at home is the long distance between the villages and health facilities. This distance makes a woman to become tired because she is already feeling heavy with the baby in the womb.

<Internals¥¥FGD¥¥Men FGD¥¥Maper_men 19032015_rev> - § 1 reference coded [3.24% Coverage]

Reference 1 - 3.24% Coverage

Some women don't deliver in health facilities because of long distances from villages to the health facility. Before Maper PHCC was opened, patients would go all the way to Rumbek State hospital, Mapurdit or Marial-lou to look for treatment. This was a big hindrance to pregnant women because they could not visit such far away facilities.

<Internals¥¥FGD¥¥Men FGD¥¥Ror bar_men 16032015_rev> - § 1 reference coded [0.99% Coverage]

Reference 1 - 0.99% Coverage

The reason why some women are not attending the hospital is the long distance from the villages to the health facility.

<Internals¥¥FGD¥¥Women FGD¥¥FGD Achiek 17032015_women> - § 5 references coded [6.43% Coverage]

Reference 1 - 0.51% Coverage

The other thing is that the hospital is very far.

Reference 2 - 1.04% Coverage

The hospital is very far from us and there are no means of transportation to take us there during labour.

Reference 3 - 1.54% Coverage

For all this time, the main hospital where pregnant women could deliver was Rumbek Hospital, and it is very far from us; we cannot reach there by walking.

Reference 4 - 1.33% Coverage

The hospital of Maper is very far from us and it is the only hospital in the whole of Rumbek North where a pregnant woman can deliver.

Reference 5 - 2.01% Coverage

I delivered at home because the hospital is far. While I was in labour pain, I was not able to

move. If the hospital was near or if there was means of transportation, I would have gone to the hospital.

<Internals¥¥FGD¥¥Women FGD¥¥FGD Biar 14032015_women> - § 5 references coded [5.05% Coverage]

Reference 1 - 1.78% Coverage

So my sisters have said everything but there are two things which we do not have; one is the hospital and the other is the market. We really stay in the bush; if you lack salt you cannot get it nearby. This is similar to going to the hospital. We don't get health care because health facilities are very far away from us.

Reference 2 - 1.16% Coverage

The other thing is that the hospital is very far. If you are in labour and about to give birth, you cannot manage to walk for a long distance. But if the hospital is near, you can be confident of reaching there.

Reference 3 - 0.74% Coverage

What we need is a hospital to be constructed near us so that when you are in urgent labour, we can rush there to be helped by doctors.

Reference 4 - 0.98% Coverage

There is nothing we know about the hospital apart from life. The only problem is that the hospital is very far from us. If the hospital was near, our lives could have improved.

Reference 5 - 0.39% Coverage

Some women deliver at home because the hospital is very far from them.

<Internals¥¥FGD¥¥Women FGD¥¥FGD Chatom Alor 13032015_women> - § 7 references coded [6.14% Coverage]

Reference 1 - 1.73% Coverage

Sometimes, even if I desire to go to hospital, there will be no way for me to reach there because it is very far. There is no another hospital which is near to us except the one of Maper. If you really want to help us, you better bring a hospital to our place so that we shall deliver without any problems.

Reference 2 - 1.20% Coverage

When you are pregnant and the hospital is very far like in Maper where pregnant mothers should deliver, there will be no way of us to go to Maper to deliver because we usually don't know the time of our delivery.

Reference 3 - 1.03% Coverage

The other day I was told by the mid-wife to return to the hospital to deliver, but I did not go. This is because the hospital is far; If were near, I could have gone to deliver there.

Reference 4 - 1.05% Coverage

I have delivered 3 children without going to the hospital because of the distance. Whenever I have problems, I go to the traditional healer because I have nowhere else to go to be helped.

Reference 5 - 0.42% Coverage

In my opinion, if the hospital were near us, we would have delivered there

Reference 6 - 0.29% Coverage

The other reason is that the hospital is very far.

Reference 7 - 0.41% Coverage

The hospital is very far otherwise, we would have gone there to deliver.

<Internals¥¥FGD¥¥Women FGD¥¥FGD Madhol Malueth 12032015_women> - § 8 references coded [6.04% Coverage]

Reference 1 - 0.67% Coverage

Sometimes you might be in labour for 3 days, if Gods helps, you deliver safely, if not, you or the child might die because the hospital is very far.

Reference 2 - 0.77% Coverage

Because we lack health facilities, during my last pregnancy, when it came to the time of my delivery, my child died before coming out and I remained sick for a long time.

Reference 3 - 0.22% Coverage

We deliver at home because the hospital is far.

Reference 4 - 0.79% Coverage

If I was staying near a hospital, I would be going there every time I am in labour. But delivering at home worries me a lot because I am used to delivering in the hospital.

Reference 5 - 0.74% Coverage

The hospital is very far away from this village and that is why we are used to delivering at home. If you bring a health center to Madhol, we will be very glad.

Reference 6 - 0.85% Coverage

In conclusion, what we need most is for us to have a health facility nearby so that if we are in labour, we can consult the nurses immediately without walking for long and wasting time.

Reference 7 - 1.04% Coverage

We prefer to deliver in the hospital, if you bring a hospital near us, we shall be going to deliver in it. Last year when I was staying in Rumbek near the hospital, I went and got treated but now I am very far from any hospital.

Reference 8 - 0.96% Coverage

Maper hospital and Kak hiospital are very far from us and so we decide not to go to hospital.

In our village, we are used to delivering at home because we cannot reach to the hospital because it is very far.

<Internals¥¥FGD¥¥Women FGD¥¥FGD Maper 19032015 women> - § 2 references coded [1.79% Coverage]

Reference 1 - 0.53% Coverage

I did not deliver in the health facility because it was very far from me.

Reference 2 - 1.26% Coverage

The hospital is very near and it has given us freedom. Even if you give birth at home, you will go to the hospital and both you and your child will be injected and be fine.

<Internals¥¥FGD¥¥Women FGD¥¥FGD Meen village 18032015_women> - § 1 reference coded [0.54% Coverage]

Reference 1 - 0.54% Coverage

The reason is that health facilities are very far from us and there is nobody that we can leave at home.

<Internals¥¥FGD¥¥Women FGD¥¥FGD Nhomleng camp2 21032015_women> - § 1 reference coded [2.89% Coverage]

Reference 1 - 2.89% Coverage

If you are in labour and are about to give birth, you cannot manage to walk for a long distance because the hospitals are very far from us. This is because we people in the cattle camps can sometimes move very far in search of good grazing grounds. After some months, we might feel that this place is not good for our cattle and so we may move to new a place in search of water and pastures for our cattle.

<Internals¥¥FGD¥¥Women FGD¥¥FGD Nhomleng campe1 21032015_women> - § 3 references coded [3.37% Coverage]

Reference 1 - 1.11% Coverage

We don’t have good care, such as going to the hospital for treatment whenever we are sick because the hospital is very far from us

Reference 2 - 1.17% Coverage

It is because our places are very far from the health facility; even if we desire to deliver in the hospital, we cannot reach the place.

Reference 3 - 1.09% Coverage

For all this time we have not been having any hospital where we could deliver except the hospitals in Warrap and Rumbek centre.

<Internals¥¥FGD¥¥Women FGD¥¥FGD Ror bar 16032015_women> - § 2 references coded [2.24% Coverage]

Reference 1 - 1.11% Coverage

The hospital of Maper is very far from us. If we are in labour, we cannot reach the hospital to deliver.

Reference 2 - 1.13% Coverage

The hospital in Maper is very far from us; that is the only hospital where a pregnant woman can deliver.

<Internals¥¥FGD¥¥Women FGD¥¥FGD Wundhiot 23032015_women> - § 2 references coded [2.00% Coverage]

Reference 1 - 1.44% Coverage

Meen Hospital and Maper Hospital are very far from us. We are actually in the middle between Rumkek and Maper hospitals. If you want to go to hospital, you can spend one day to reach there.

Reference 2 - 0.56% Coverage

Sometime we give up in going to hospital because the hospital is very far.

<Internals¥¥KIIs¥¥KII CHD M_E> - § 1 reference coded [4.56% Coverage]

Reference 1 - 4.56% Coverage

The condition of the far place like Alor is not good. We recently opened a clinic in Nanimba, but all along this place has not been having a health facility. And it is a large place. Like now if you go and tell women to deliver in a health facility they will not come because of distance; for example from here to Alor is three hours on foot. Pregnant women cannot walk from such a far place.

<Internals¥¥KIIs¥¥KII CHW Maper 4> - § 1 reference coded [2.16% Coverage]

Reference 1 - 2.16% Coverage

One reason is the distance as I have mentioned before.

<Internals¥¥KIIs¥¥KII TBA 5 Malueth> - § 2 references coded [3.88% Coverage]

Reference 1 - 1.91% Coverage

People complain that they stay very far from a health facility

Reference 2 - 1.97% Coverage

The hospital is very far. Some villages are very far from Maper.

<Internals¥¥KIIs¥¥KII TBA1> - § 1 reference coded [1.40% Coverage]

Reference 1 - 1.40% Coverage

The distance is also the other issue.

<Internals¥¥KIIs¥¥KII TBA2> - § 1 reference coded [8.46% Coverage]

Reference 1 - 8.46% Coverage

The problem is distance. We don't have a road. Because of floods after rain, even if you are in labour and want to go to the health facility, you will have no way of reaching there.

<Internals¥¥KIIs¥¥KII TBA4> - § 1 reference coded [7.92% Coverage]

Reference 1 - 7.92% Coverage

The problem is the distance, if a woman is suffering in Malueth or Madol, and it is during the rainy season, there is no way a vehicle can reach there to go and pick her.

<Internals¥¥KIIs¥¥KII_Malueth Chief 1> - § 1 reference coded [3.59% Coverage]

Reference 1 - 3.59% Coverage

The other reason is that the hospital is very far from us and we don't know when the woman will deliver.

Transport means availability

<Internals¥¥FGD¥¥Women FGD¥¥FGD Achiek 17032015_women> - § 3 references coded [3.11% Coverage]

Reference 1 - 1.04% Coverage

The hospital is very far from us and there are no means of transportation to take us there during labour.

Reference 2 - 1.22% Coverage

The other reason is that we don't have means of transportation which a pregnant woman can use to go to the health facility.

Reference 3 - 0.85% Coverage

If we are in labour pain, there is not means of transport to take us to the hospital.

<Internals¥¥FGD¥¥Women FGD¥¥FGD Maper 19032015 women> - § 1 reference coded [0.73% Coverage]

Reference 1 - 0.73% Coverage

When in labour, there is no time to go to the hospital because there are no means of transportation.

<Internals¥¥FGD¥¥Women FGD¥¥FGD Meen village 18032015_women> - § 1 reference coded [0.59% Coverage]

Reference 1 - 0.59% Coverage

The other thing is that we who are staying in the village lack means of transportation to take us to the hospital.

<Internals¥¥FGD¥¥Women FGD¥¥FGD Nhomleng campe1 21032015_women> - § 2 references coded [1.79% Coverage]

Reference 1 - 1.48% Coverage

The only problem is that we don't know the time when we shall deliver and when labour starts, we are not able to reach the hospital because we lack means of transportation.

Reference 2 - 0.31% Coverage

There is no means of transportation.

<Internals¥¥KIIs¥¥KII CHD CMO> - § 1 reference coded [3.60% Coverage]

Reference 1 - 3.60% Coverage

Another reason is that some stay in the cattle camp and some are staying very far, and without transportation means they cannot reach the health facility. And you know this place doesn't have means of transportation, even if you have your money, you cannot facilitate yourself.

<Internals¥¥KIIs¥¥KII CHW Maper 1> - § 1 reference coded [3.35% Coverage]

Reference 1 - 3.35% Coverage

And there is no means of transport to come easily to the health facility.

<Internals¥¥KIIs¥¥KII TBA 5 Malueth> - § 1 reference coded [2.89% Coverage]

Reference 1 - 2.89% Coverage

The woman cannot reach the hospital because she is in labour and there is no transport means.

<Internals¥¥KIIs¥¥KII TBA Meen> - § 1 reference coded [7.99% Coverage]

Reference 1 - 7.99% Coverage

The other problem is lack of transport. During the rainy season, there are floods and that discourages women from coming here.

<Internals¥¥KIIs¥¥KII_Malueth Chief 2> - § 1 reference coded [5.87% Coverage]

Reference 1 - 5.87% Coverage

Even if the vehicle is not there, we call men to put that woman on their shoulders and take her to the health facility.

Floods and poor roads

<Internals¥¥FGD¥¥Women FGD¥¥FGD Achiek 17032015_women> - § 1 reference coded [0.96% Coverage]

Reference 1 - 0.96% Coverage

Our roads are usually flooded during the rainy season and so we cannot reach the health facility.

<Internals¥¥FGD¥¥Women FGD¥¥FGD Chatom Alor 13032015_women> - § 3 references coded [3.11% Coverage]

Reference 1 - 0.50% Coverage

The worst thing is that during the wet season all roads will be closed because of floods.

Reference 2 - 0.99% Coverage

Even now, we doubt that you will really help us because our road is not okay. During the wet season, there will be no way for you to come with your help because of the flood.

Reference 3 - 1.62% Coverage

During the last dry season when I was pregnant, I went to Maper hospital. I was injected by the white lady who told me to go back during the wet season. But during the rainy season, there were floods and I could not go back again because I don’t know how to swim, so I delivered at home.

<Internals¥¥FGD¥¥Women FGD¥¥FGD Meen village 18032015_women> - § 1 reference coded [0.48% Coverage]

Reference 1 - 0.48% Coverage

I had decided to deliver in the hospital but it started raining and the road became flooded.

<Internals¥¥FGD¥¥Women FGD¥¥FGD Nhomleng campe1 21032015_women> - § 1 reference coded [0.85% Coverage]

Reference 1 - 0.85% Coverage

Sometimes during the wet season and all roads are flooded, there is no way of going to the hospital.

<Internals¥¥FGD¥¥Women FGD¥¥FGD Wundhiot 23032015_women> - § 3 references coded [2.91% Coverage]

Reference 1 - 0.77% Coverage

We delivered at home because of floods. When it has flooded, we cannot manage to walk to the hospital.

Reference 2 - 1.07% Coverage

I did not go to the hospital during my last pregnancy because there was too much water everywhere; you could not get any dry place to step on

Reference 3 - 1.07% Coverage

The road is bad with holes all over and we are not even able to go and buy salt and soap for our babies, leave alone walking to the hospital.

<Internals¥¥KIIs¥¥KII CHD M_E> - § 1 reference coded [1.47% Coverage]

Reference 1 - 1.47% Coverage

During the rainy season, women cannot move from far place to here because the roads are flooded and there is no transport means

<Internals¥¥KIIs¥¥KII TBA2> - § 2 references coded [15.28% Coverage]

Reference 1 - 4.63% Coverage

When the road is not well, they cannot reach the hospital, but when the road is good they can come.

Reference 2 - 10.65% Coverage

We need the government to make roads so that we can be able to go the villages easily. We the people who have been trained can go. But now there is no way, even if you train us, and there is no road, I think we still cannot help

<Internals¥¥KIIs¥¥KII TBA4> - § 1 reference coded [33.01% Coverage]

Reference 1 - 33.01% Coverage

The reason why I mentioned about the road is that during the last rainy season, five women died because of lack roads and there was no way for the vehicle to go and pick them. One of the women bled a lot after delivery and she died. When people reached there with a motor bike, they found her already dead and they just brought the child whom we referred to Rumbek. Some children also die with their mothers. So we really need this road to be made. Even if you train us, and there is no way to reach that far place, we will still be unable to help. The women died in Meen Payam. There is another village called Obuoth, where a young girl who had been married at a pride price of 100 cows died during delivery of her first child.

<Internals¥¥KIIs¥¥KII_Malueth Chief 2> - § 1 reference coded [10.12% Coverage]

Reference 1 - 10.12% Coverage

This month of March is good, when it comes to the month of August, there will be no way a car can come here because of floods. During that time, we put our ladies on our shoulders to take them to the hospital.

Referrals

<Internals¥¥KIIs¥¥KII CHW Alor> - § 2 references coded [6.33% Coverage]

Reference 1 - 2.90% Coverage

I tell the mother go to Maper. If she can walk and get there she can go if not, what can I do?. I never call the ambulance because we do not have any means of communication to call the ambulance. This radio is for the Payam and there is nobody who sits here.

Reference 2 - 3.43% Coverage

For a woman who is not able to walk, I tell the community that if you are able to take her, you take her on your shoulders up to the health facility in Maper. If there is nobody to take her, I send somebody or even I go by myself to Maper PHCC to ask for the ambulance. I go to Maper walking on my foot.

<Internals¥¥KIIs¥¥KII CHW Meen PHCU> - § 1 reference coded [16.18% Coverage]

Reference 1 - 16.18% Coverage

Sometimes it is difficult to refer because there is no communication. If the car is around, then I can call it. If there is no car, I go to Maper on foot to call the ambulance. It takes me 3 hours to go to Maper walking. Sometimes when the rains come, it is more difficult. There is no radio and we don’t have a means of transport

<Internals¥¥KIIs¥¥KII_Malueth Chief 1> - § 1 reference coded [2.46% Coverage]

Reference 1 - 2.46% Coverage

We lack a road, during the wet season; we can not reach the hospital.

<Internals¥¥KIIs¥¥KII_Payam admin Meen> - § 1 reference coded [6.98% Coverage]

Reference 1 - 6.98% Coverage

There was a woman who was bleeding for more than an hour here. The CHW was troubled about what to do. He was walking up and down looking for a solution and there was no means of communication for him to communicate with you.

Costs

<Internals¥¥FGD¥¥Men FGD¥¥Maper_men 19032015_rev> - § 1 reference coded [3.67% Coverage]

Reference 1 - 3.67% Coverage

The disadvantage of delivering in a health facility is the requirement that a woman pays money for her delivery. Sometimes a poor delivering woman does not have money and this can be a headache to her. This is also bad for a poor delivering woman because she will not have any

money to buy flour for making her porridge. After paying money in the hospital, she will remain penniless.

<Internals¥¥FGD¥¥Men FGD¥¥Meen_men 18032015_rev> - § 1 reference coded [1.63% Coverage]

Reference 1 - 1.63% Coverage

One disadvantage is the charging of money when your wife delivers in a hospital. This can prevent poor women from delivering in the hospital.

<Internals¥¥FGD¥¥Women FGD¥¥FGD Achiek 17032015_women> - § 1 reference coded [1.41% Coverage]

Reference 1 - 1.41% Coverage

We are very poor and we cannot afford to pay money in the maternity in Rumbek. Mothers are asked to pay money at the main hospital in Rumbek.

<Internals¥¥FGD¥¥Women FGD¥¥FGD Madhol Malueth 12032015_women> - § 2 references coded [0.70% Coverage]

Reference 1 - 0.40% Coverage

If we go to the hospital, we are asked for money and we do not have enough money to pay.

Reference 2 - 0.30% Coverage

What we dislike most is being asked for money that we don't have.

<Internals¥¥FGD¥¥Women FGD¥¥FGD Maper 19032015 women> - § 1 reference coded [1.76% Coverage]

Reference 1 - 1.76% Coverage

When I was pregnant, I decided to deliver in the hospital but when I went to the main hospital in Rumbek, I was asked to pay money in the maternity. By then this hospital in Maper had not yet been opened and so I came back to deliver at home.

<Internals¥¥FGD¥¥Women FGD¥¥FGD Meen village 18032015_women> - § 1 reference coded [0.89% Coverage]

Reference 1 - 0.89% Coverage

What I dislike about the hospital is that, when we go to get help, after delivery the mother is asked to pay money, but we don’t have money; we just go there to get help.

Influence of the socio-cultural context and conflict

Insecurity

<Internals¥¥FGD¥¥Women FGD¥¥FGD Achiek 17032015_women> - § 1 reference coded [2.35% Coverage]

Reference 1 - 2.35% Coverage

> Our place is also in the middle of enemies who frequently attack us. Some of us fear that if we go to deliver in the hospital and the enemy comes to attack our children in our absence, there will be nobody to lead them to a hiding place

<Internals¥¥FGD¥¥Women FGD¥¥FGD Nhomleng camp2 21032015_women> - § 1 reference coded [1.44% Coverage]

Reference 1 - 1.44% Coverage

Due to the political and economic problems, we have so many challenges such as hunger and fighting with the [*neighbouring tribe*] every day. The [*neighbouring tribe*] might attack children if we leave them behind and go to the hospital.

<Internals¥¥KIIs¥¥KII CHW Alor> - § 1 reference coded [1.05% Coverage]

Reference 1 - 1.05% Coverage

We had insecurity problem here and the people were evacuated to a place where they can be safe

Influence of husband/male partner

<Internals¥¥FGD¥¥Men FGD¥¥Achiek_men 17032015_rev> - § 4 references coded [8.64% Coverage]

Reference 1 - 2.47% Coverage

The health facility provides information about the days or months to the delivery day. This can help the husband and family to decide where the woman should give birth.

Reference 2 - 2.34% Coverage

The other reason is that our community ignores women. Some men wonder why women should always cry during labour. They take lightly the pain of pregnant women.

Reference 3 - 1.19% Coverage

The one who decides where a woman should give birth is the husband of that woman.

Reference 4 - 2.65% Coverage

A man decides to let his wife deliver in the hospital or at home. He also decides to prepare the delivery requirements such as sleeping place, pieces of clothes for the baby and food.

<Internals¥¥FGD¥¥Men FGD¥¥Maper_men 19032015_rev> - § 3 references coded [4.68% Coverage]

Reference 1 - 1.95% Coverage

The husband of the pregnant woman is the right person to decide where she will give birth. The man is the one who married her and he will decide where she will give birth and will provide all she needs.

Reference 2 - 1.24% Coverage

A man can decide where his pregnant woman should give birth after realising that the woman is having difficulties in delivering.

Reference 3 - 1.49% Coverage

There are two types of men. There are those who care about where their wives should give birth and there are those that don’t care about place of delivery.

<Internals¥¥FGD¥¥Men FGD¥¥Meen_men 18032015_rev> - § 2 references coded [1.24% Coverage]

Reference 1 - 0.56% Coverage

Some people force their wives to deliver at home

Reference 2 - 0.68% Coverage

It is the man who decides where a woman should give birth.

<Internals¥¥FGD¥¥Men FGD¥¥Ror bar_men 16032015_rev> - § 1 reference coded [0.72% Coverage]

Reference 1 - 0.72% Coverage

The husband decides where a woman should give birth; either in the hospital or at home.

<Internals¥¥FGD¥¥Women FGD¥¥FGD Achiek 17032015_women> - § 1 reference coded [1.10% Coverage]

Reference 1 - 1.10% Coverage

Our husbands restrict us to deliver in the hospital saying that we have to deliver at home as our mothers did.

<Internals¥¥FGD¥¥Women FGD¥¥FGD Madhol Malueth 12032015_women> - § 7 references coded [10.58% Coverage]

Reference 1 - 2.72% Coverage

I went to Kak yesterday and I was told to give birth in the hospital. When I passed this message to my husband, he said there was no way I would go to the hospital again. He said "who will remain with your children again tomorrow"? If I decide by myself to go to the hospital against his will, he will become violent with me. He will start saying that I have become rebellious.

If you plan by yourself to go to the hospital, your husband will get annoyed with you and will say that you like doing what you have decided to do not what he has decided. This one will bring a conflict in the family.

Reference 2 - 0.54% Coverage

Because it is the man who allows you to go to the hospital, how can you go to the hospital if your husband has refused.

Reference 3 - 1.12% Coverage

Now my sisters, you see that pregnant woman, she was told to go to the hospital. And if she tells her husband that she is going to hospital, the husband will tell her that she is not going anywhere. It is true that women are under the control of men.

Reference 4 - 3.18% Coverage

However, if the hospital is very near, even if a man says you can't go, you can decide by yourself to escape without his knowledge. Doctors can test you and give you medicine until you deliver there. But now the hospital is very far and if you decide to go and you have very little children, who do you think will take care of those children. If the hospital is near, even if children are young to be left alone, you can go and come back and find them still okay.

Some men would say that "you cannot go because of your child". If you don't agree, they will beat you up. So if you bring the hospital near us, nobody will restrict you from going because the hospital will be near and it can be seen

Reference 5 - 1.50% Coverage

We know the advantage delivering in hospital but our men are the ones who refuse to allow us to go to the hospital. Everybody says that the hospital is good but you know women stay under men's control. Even if you told him what you want, if he doesn't agree with what you have said, he will not allow you to go to the hospital.

Reference 6 - 0.75% Coverage

For me, I was told to give birth in the hospital. When I told my husband about this, he did not allow me to go. Instead he asked me who I would leave my child with.

Reference 7 - 0.77% Coverage

It is the man who insists that a woman should deliver at home. Men say that their mothers gave birth to them at home and they wonder why their wives can't do likewise.

<Internals¥¥FGD¥¥Women FGD¥¥FGD Maper 19032015 women> - § 1 reference coded [2.78% Coverage]

Reference 1 - 2.78% Coverage

During my first pregnancy, I told my husband that I need to deliver in a health facility but he refused to allow me; saying that delivering in the hospital was not good because he did not want

me to be seen by men when I am delivering. When I came back to deliver at home, I gave birth to twins and both of them died. I remained regretting for not having delivered in the hospital.

<Internals¥¥FGD¥¥Women FGD¥¥FGD Meen village 18032015_women> - § 1 reference coded [1.81% Coverage]

Reference 1 - 1.81% Coverage

The husband is the one who decides where a woman should give birth. Even if a woman has decided to deliver in the hospital that husband will say “no, you are just going to roam there, you must deliver here. Whom will you leave your children with if you decide to go and deliver in the hospital?” Our husbands choose the place where we should deliver.

<Internals¥¥FGD¥¥Women FGD¥¥FGD Nhomleng camp2 21032015_women> - § 2 references coded [3.17% Coverage]

Reference 1 - 1.58% Coverage

Last month I was pregnant and my months were over. I was admitted in the hospital because I was having abdominal pains swollen legs. But my husband said that I cannot deliver in the hospital and so I went to deliver at home.

Reference 2 - 1.59% Coverage

Sometimes your husband will not allow you to deliver in the hospital. He will say “whom will you leave your children and animals with? Do you remember I married you after paying many cows? You don’t listen to me, I am your husband”

<Internals¥¥FGD¥¥Women FGD¥¥FGD Wundhiot 23032015_women> - § 2 references coded [2.66% Coverage]

Reference 1 - 1.63% Coverage

When I was pregnant, I went to the hospital and was told that I must deliver in a hospital. However, when I came to tell my husband and my mother in -law, they refused and insisted that I have to deliver at home.

Reference 2 - 1.04% Coverage

For you my sister from Rumbek, your husband can allow you to go to the hospital but here, our men cannot allow us to go to the hospital.

<Internals¥¥KIIs¥¥KII CHD M_E> - § 1 reference coded [4.06% Coverage]

Reference 1 - 4.06% Coverage

Husbands who know that the health facility is good can push their wives to deliver in the health facility. But this will depend on the condition of the woman. Is she is normal, he can decide to stay at home. But if he sees that the woman has a problem and delivery is becoming very difficult, that is when they will try to come to the health facility.

<Internals¥¥KIIs¥¥KII CHW Aciek PHCU> - § 1 reference coded [4.97% Coverage]

Reference 1 - 4.97% Coverage

Some men are not educated. If the mother wants to deliver in the health facility, they say no because for all this time, their mothers used to deliver at home.

<Internals¥¥KIIs¥¥KII CHW Maper 3> - § 1 reference coded [8.00% Coverage]

Reference 1 - 8.00% Coverage

If she say that she wants to go to the hospital to deliver, the husband will refuse to come with her because he is uneducated and doesn't know the benefit of delivering in the health facility.

<Internals¥¥KIIs¥¥KII TBA 5 Malueth> - § 2 references coded [15.77% Coverage]

Reference 1 - 5.85% Coverage

Some of the husbands restrict their wives from delivering in the health facility; this is a common thing. They will say "you just deliver at home, what are you going to get in the hospital?"

Reference 2 - 9.92% Coverage

Our women are now changing a bit because they come to the hospital to be tested and when they do so, the TBA has a role to inform her to come to deliver in the health facility. But her husband can refuse, saying that the woman should not go because there is no soap in the hospital and there is nothing she will be given.

<Internals¥¥KIIs¥¥KII TBA1> - § 1 reference coded [9.69% Coverage]

Reference 1 - 9.69% Coverage

Women rely on the husband. If the woman is not feeling well during pregnancy, she has to ask for permission from the husband to be allowed to visit the health facility. She cannot just decide on her own to go to the hospital, she has to discuss with the husband.

<Internals¥¥KIIs¥¥KII TBA3> - § 1 reference coded [10.34% Coverage]

Reference 1 - 10.34% Coverage

Actually it is the man. For instance if a TBA sees that a woman who has come for ANC and is not able to deliver a home, she goes directly to the husband and tell him that his wife is like this, she needs to deliver in the health facility.

<Internals¥¥KIIs¥¥KII TBA4> - § 1 reference coded [10.08% Coverage]

Reference 1 - 10.08% Coverage

Our husbands don’t know the benefit of the hospital. They just decide for you to deliver at home. Even if we advise the mother to come and deliver and the man declines to allow her to come, there is nothing she can do.

<Internals¥¥KIIs¥¥KII TBA2> - § 1 reference coded [9.77% Coverage]

Reference 1 - 9.77% Coverage

If a woman is having a difficult delivery, the husband can take her to the hospital because the delivery is difficult. But if it is normal, she can deliver at home. It is the man who decides, not the woman.

Preparedness for childbirth

Lack of preparations was attributed to poverty, long distance to trading centres where items could be purchased and lack of education.

<Internals¥¥FGD¥¥Women FGD¥¥FGD Achiek 17032015_women> - § 1 reference coded [1.41% Coverage]

Reference 1 - 1.41% Coverage

We women in this village have nothing to prepare because the town is very far from us and we don’t have enough resources to make preparations.

<Internals¥¥FGD¥¥Women FGD¥¥FGD Biar 14032015_women> - § 4 references coded [4.41% Coverage]

Reference 1 - 1.00% Coverage

We don’t prepare for delivery because we have nothing to keep for delivery. Now you have seen our place deep here in the bush; we have nothing to eat leave alone prepare for delivery.

Reference 2 - 0.86% Coverage

Another problem is that delivery just comes abruptly like a fruit falling from the tree. So some people deliver before having prepared anything for delivery.

Reference 3 - 1.23% Coverage

For us, we don’t know the day of delivery; even the husband doesn’t know because we are all uneducated people. But in the hospital, the doctors know even though they are not our husbands. They know because they are educated.

Reference 4 - 1.31% Coverage

Nobody decides where you should give birth, but when the time comes, whether you are in the field cultivating or in the compound sweeping, and the baby is about to come, you just call the TBA who may not even be prepared for your delivery.

<Internals¥¥FGD¥¥Women FGD¥¥FGD Chatom Alor 13032015_women> - § 3 references coded [2.55% Coverage]

Reference 1 - 0.79% Coverage

It is not that we don’t know preparations that a mother should make before birth. Only that we don’t have resources for preparing ourselves.

Reference 2 - 1.23% Coverage

Nobody takes care for us unlike in other areas. The husband is supposed to take the responsibility over his wife’s preparations by for example providing soap and small pieces of cloth for the baby, sugar and good food.

Reference 3 - 0.53% Coverage

We don’t prepare anything before delivery because our place is far from town and we are poor.

<Internals¥¥FGD¥¥Women FGD¥¥FGD Madhol Malueth 12032015_women> - § 2 references coded [1.56% Coverage]

Reference 1 - 0.77% Coverage

There is nothing like preparations during pregnancy in our place. We don’t make any preparations until when we deliver; this is when something to eat will be gathered.

Reference 2 - 0.79% Coverage

It is not that we don’t know what preparations a mother has to make before birth. It is just that there is no way that we can get the items we need such as soap and clothes.

<Internals¥¥FGD¥¥Women FGD¥¥FGD Maper 19032015 women> - § 3 references coded [9.36% Coverage]

Reference 1 - 7.05% Coverage

Last time when I was pregnant, I asked my husband to buy me pieces of cloth, soap, sugar, cups, bed sheet and a baby carrier and he said “why should I buy for you such items before you give birth? Who told you that you should buy those things before delivering? Those things will be bought after you deliver. Long time ago, my mother never used to ask for such nonsense. Where on earth can the cloth of the unborn baby be bought while it is still in the womb? Who knows whether the baby will come out alive or dead?” The husband cannot make and preparations for the wife unless he is educated.

We don’t prepare anything before delivery. This is because some babies die before spending a day. Now imagine if you had prepared inside your house, you will regret for having bought those things for nothing because your baby will not be there. For this reason, some women like me don’t like such a shame. The only thing you do is to ask God to help you during delivery.

Reference 2 - 1.50% Coverage

Some women who deliver and their babies die will be there with their preparations and other women will start saying that they don’t want to be like so and so who prepared and received no child on her hands.

Reference 3 - 0.81% Coverage

The other reason why we don’t deliver in a health facility is that we don’t know the expected day of delivery.

<Internals¥¥FGD¥¥Women FGD¥¥FGD Meen village 18032015_women> - § 2 references coded [1.38% Coverage]

Reference 1 - 0.87% Coverage

The other thing is that, our delivery time comes abruptly without us knowing. Even if we decide to deliver in the hospital, it is when we are already in labour pains.

Reference 2 - 0.52% Coverage

We deliver at home because we are not educated to know the day of delivery. It just comes suddenly.

<Internals¥¥FGD¥¥Women FGD¥¥FGD Nhomleng camp2 21032015_women> - § 1 reference coded [1.21% Coverage]

Reference 1 - 1.21% Coverage

In the traditional way, we prepare nothing. We stay like that without preparing for anything until delivery. The baby is delivered like that on the floor or under a tree.

<Internals¥¥FGD¥¥Women FGD¥¥FGD Nhomleng campe1 21032015_women> - § 4 references coded [2.45% Coverage]

Reference 1 - 0.69% Coverage

There are no preparations we make before birth because we are living in the bush.

Reference 2 - 0.60% Coverage

When we are pregnant, we don’t know the exact timing of our delivery.

Reference 3 - 0.56% Coverage

Sometimes, labour pains just come abruptly without our knowledge.

Reference 4 - 0.61% Coverage

The other thing is that we don’t know the expected time of delivery.

<Internals¥¥FGD¥¥Women FGD¥¥FGD Ror bar 16032015_women> - § 5 references coded [6.67% Coverage]

Reference 1 - 0.84% Coverage

There are no preparations we make during pregnancy because we lack resources.

Reference 2 - 2.41% Coverage

A pregnant woman waits until the day of delivery without making any preparations. For example, I delivered just 10 days ago and I lack soap for bathing the baby. I don’t even have pieces of clothes for my baby and good food.

Reference 3 - 0.84% Coverage

None of us knows the day of our delivery. We just wait until that time comes.

Reference 4 - 1.03% Coverage

The other reason is that, we don’t know our exact day of delivery. Delivery time comes abruptly.

Reference 5 - 1.55% Coverage

Even if we have decided to deliver in the health facility, some of us are not aware of the day of delivery, and the health facility is very far.

<Internals¥¥FGD¥¥Women FGD¥¥FGD Wundhiot 23032015_women> - § 2 references coded [2.54% Coverage]

Reference 1 - 1.26% Coverage

If it happens that the woman is already in labour and we have not made any preparations, there is no time to prepare for anything because the town is very far from us.

Reference 2 - 1.28% Coverage

Another reason why we do not go to hospital is that we do not know the day when the baby will come out. If we knew the day of delivery, we would be preparing ourselves.

<Internals¥¥KIIs¥¥KII CHD CMO> - § 1 reference coded [8.77% Coverage]

Reference 1 - 8.77% Coverage

It depends on where the labour starts. How many women deliver on the road here? It is not a decision by somebody to say you deliver here. People did not know the importance of the hospital, they delivered anywhere. But if we give this information to them, they can consider about delivering in the health facility. They should get the information otherwise they will deliver anywhere including at home, in cattle camp and along the road; that is why you hear some people being called Makuer, Mawut, Maiwit. Makuer was born on the road, Mawut is somebody born in the cattle camp, Muiwit is somebody born along the river when the mother went to fetch water and delivered there.

<Internals¥¥KIIs¥¥KII CHW Maper 1> - § 1 reference coded [6.13% Coverage]

Reference 1 - 6.13% Coverage

They don’t know the expected day of delivery and so labour starts when they are not prepared and have no time to go to the health facility.

<Internals¥¥KIIs¥¥KII_Malueth Chief 1> - § 1 reference coded [6.28% Coverage]

Reference 1 - 6.28% Coverage

Delivery comes abruptly when nobody is expecting. Nobody knows the expected time of delivery. We don’t tell our women not to deliver in the health facility. Labour just comes abruptly.

Women’s domestic chores

<Internals¥¥FGD¥¥Women FGD¥¥FGD Achiek 17032015_women> - § 1 reference coded [1.98% Coverage]

Reference 1 - 1.98% Coverage

> The husband can ask the wife to tell him who she will leave the children with if she decides to go and deliver in the hospital. With no one to leave children with, the wife will just deliver at home.

<Internals¥¥FGD¥¥Women FGD¥¥FGD Biar 14032015_women> - § 1 reference coded [0.65% Coverage]

Reference 1 - 0.65% Coverage

If you have a desire to go to the hospital for yourself or your baby, you will have nobody to leave your husband with.

<Internals¥¥FGD¥¥Women FGD¥¥FGD Madhol Malueth 12032015_women> - § 2 references coded [1.07% Coverage]

Reference 1 - 0.70% Coverage

The reason we don’t go to hospital is because there is no hospital which is near to us. If we decide to go, there is nobody to leave our children with.

Reference 2 - 0.37% Coverage

Also, your children will remain with no one if you decide to go to the hospital.

<Internals¥¥FGD¥¥Women FGD¥¥FGD Maper 19032015 women> - § 1 reference coded [1.82% Coverage]

Reference 1 - 1.82% Coverage

For my case, I am the only woman at home. There is nobody else there to help me. Therefore, my husband cannot allow me to deliver in a health facility because there will be nobody to cook for children and all domestic work at home will remain undone.

<Internals¥¥FGD¥¥Women FGD¥¥FGD Meen village 18032015_women> - § 1 reference coded [0.90% Coverage]

Reference 1 - 0.90% Coverage

Some of us stay home alone with nobody there to help us. There is no way we can leave home and go to deliver in the hospital because there is nobody that we can leave at home

<Internals¥¥FGD¥¥Women FGD¥¥FGD Nhomleng camp2 21032015_women> - § 1 reference coded [0.76% Coverage]

Reference 1 - 0.76% Coverage

We deliver here in the cattle camp because we are the ones to take care of all calves (in the cattle camp).

<Internals¥¥FGD¥¥Women FGD¥¥FGD Nhomleng campe1 21032015_women> - § 1 reference coded [2.66% Coverage]

Reference 1 - 2.66% Coverage

In our place here we are preoccupied with our work and birth can come suddenly without knowing the time. For example, when my sister was in labour pain, we were trying to take her to the hospital but she just delivered on the road going to the hospital because we just found her while she was already in labour.

<Internals¥¥FGD¥¥Women FGD¥¥FGD Ror bar 16032015_women> - § 1 reference coded [1.54% Coverage]

Reference 1 - 1.54% Coverage

When our children are still very young, there is nobody to leave them with if we want to go to the hospital, and that is why we deliver at home.

<Internals¥¥FGD¥¥Women FGD¥¥FGD Wundhiot 23032015_women> - § 4 references coded [5.11% Coverage]

Reference 1 - 1.88% Coverage

Sometimes, our husbands would say that they need to take cattle to a place where there is enough pasture and water. We are then left behind to take care of the home and with nobody else to leave children with in case we want to go to the hospital.

Reference 2 - 1.13% Coverage

The reason we don't go the hospital is because of our children. There is nobody to leave with them at home and that is why we decide not to hospital.

Reference 3 - 1.14% Coverage

The reason we do not go to hospital is that there will be nobody to leave at home to take cate of our domestic animals like cattle, goats and chicken.

Reference 4 - 0.95% Coverage

Now the rainy season is almost coming and we will be cultivating and shall have no time to go to hospital even if we are sick.

<Internals¥¥KIIs¥¥KII CHW Alor> - § 1 reference coded [2.46% Coverage]

Reference 1 - 2.46% Coverage

The health facility is too far. A woman who lives in a far place cannot go to the hospital because she cannot leave her children behind. If there is any kind of running, there will be nobody to take care of the children.

Influence of tradition and culture

<Internals¥¥FGD¥¥Men FGD¥¥Maper_men 19032015_rev> - § 1 reference coded [1.55% Coverage]

Reference 1 - 1.55% Coverage

Traditional beliefs also play a part in this because some women belief in some gods. They believe that they need to take the child to the preventer of diseases.

<Internals¥¥FGD¥¥Men FGD¥¥Meen_men 18032015_rev> - § 2 references coded [8.25% Coverage]

Reference 1 - 3.31% Coverage

There are ways in which delivering in a health facility is good. Traditionally, babies were born at home and stayed for a long time without being washed. This concept has remained in the memory of our community. This practice makes some women not to go to a health facility for delivery.

Reference 2 - 4.94% Coverage

If the husband suspects that his wife committed adultery, he will insist on her delivering at home so that she can mention the name of the man with whom she committed adultery. If the woman committed adultery, the baby will not come out until she mentions the name of the father. Usually the midwife would hide this information from the husband and because of this men do not allow their wives to deliver in the health facility.

<Internals¥¥FGD¥¥Women FGD¥¥FGD Biar 14032015_women> - § 2 references coded [2.05% Coverage]

Reference 1 - 0.77% Coverage

Long time ago, our great grandmothers never delivered in the hospital, but delivered at

home. For this reason, we prefer to deliver at home.

Reference 2 - 1.28% Coverage

The other reason why delivering with the help of TBAs is good is that the placenta is handled properly; it is washed, put in the tin and covered. A hole is then dug and it is buried. This is what I like most about delivering at home.

<Internals¥¥FGD¥¥Women FGD¥¥FGD Chatom Alor 13032015_women> - § 2 references coded [2.76% Coverage]

Reference 1 - 1.72% Coverage

The goodness of delivering at home is that afterbirth, water is warmed and put in a bowl and the woman takes a bath. After bath, the mother takes the baby and stays with the baby without washing him/her. Also, boiled is brought and the mother takes the milk when it is still warm so as to warm the stomach.

Reference 2 - 1.04% Coverage

A woman who is in labour pain should not be given water to drink even if the labour lasts for 3 days. It is believed that if the mother takes water, the baby will not come out quickly.

<Internals¥¥FGD¥¥Women FGD¥¥FGD Madhol Malueth 12032015_women> - § 4 references coded [5.99% Coverage]

Reference 1 - 1.49% Coverage

Traditionally, women are the ones who help us. They hold you at the shoulders as you squat during delivery and the TBA will be waiting with open hands below to receive the baby. It is yourself who will be responsible for pushing the child, not somebody else. Even if it is three days, people will be waiting for you to deliver.

Reference 2 - 1.33% Coverage

Traditionally, the women give birth at home. The baby is then covered with a piece of cloth and the umbilical cord is cut and the placenta is separated. After that somebody will go and dig a hole and burry the placenta. They then go and look for a small stick with two branches and put there.

Reference 3 - 2.22% Coverage

Traditionally, a woman would spend ten days without bathing; eating good food and being given warm water. Both the mother and the baby will not shower until after 10 days or two weeks. After two weeks you will shower and also your baby will be washed; that is the traditional way

of handling a woman who has delivered and is considered to be safe. But delivering in the hospital is different. If I deliver now in the hospital, I will be bathed immediately and my baby too will be washed.

Reference 4 - 0.94% Coverage

Traditionally, it is believed that if the placenta is not buried, a woman will not give birth again. We fear that if we go to the hospital the placenta will not be handled well and we may not deliver gain.

<Internals¥¥FGD¥¥Women FGD¥¥FGD Maper 19032015 women> - § 1 reference coded [0.82% Coverage]

Reference 1 - 0.82% Coverage

In our culture, when a woman delivered, the placenta is buried and the child remains for 7 days without bathing.

<Internals¥¥FGD¥¥Women FGD¥¥FGD Meen village 18032015_women> - § 1 reference coded [0.66% Coverage]

Reference 1 - 0.66% Coverage

Our grandmothers never went to hospitals but they gave birth normally and so we shall follow the way they used to give birth.

<Internals¥¥FGD¥¥Women FGD¥¥FGD Nhomleng camp2 21032015_women> - § 2 references coded [3.80% Coverage]

Reference 1 - 1.46% Coverage

I like the hospital because there is no specific day on which your baby should be bathed, but here in the cattle camp, we are told that we need to spend ten days before you and your child can take a shower

Reference 2 - 2.33% Coverage

Some women deliver at home because it is our tradition. We are still following the way of our grandmothers who always delivered at home and not in the hospital. Our grandmothers say that they have ten children whom they delivered at home without anything happening to them. We know that God is our only helper during delivery.

<Internals¥¥KIIs¥¥KII CHD CMO> - § 1 reference coded [2.87% Coverage]

Reference 1 - 2.87% Coverage

Sometimes it is culture. They don’t like to be exposed where there are other people. Second they don’t like the placenta to be put into waste management. For them, there is certain way they handle the placenta and bury it.

<Internals¥¥KIIs¥¥KII TBA 5 Malueth> - § 2 references coded [13.46% Coverage]

Reference 1 - 11.40% Coverage

If you suspect that your wife slept with somebody else, the truth can manifest during delivery. The child cannot come out until the woman says whom she slept with. It is until she has said whom she slept with that the child will come out. If she doesn’t say, the child will not come out. Then the husband will go and look for that man who played that act with his wife.

Reference 2 - 2.06% Coverage

If woman delivers at home, the husband is usually around to watch.

<Internals¥¥KIIs¥¥KII_Payam admin Meen> - § 1 reference coded [7.63% Coverage]

Reference 1 - 7.63% Coverage

In the past people used to say that it is very simple for a woman to deliver, that is what is still in their heads. People still think that delivery is a simple thing and there is no need to go to the hospital. There are no other cultural factors

Perceptions of pregnancy and childbirth

Benefits of institutional childbirth unknown

<Internals¥¥FGD¥¥Men FGD¥¥Maper_men 19032015_rev> - § 2 references coded [2.99% Coverage]

Reference 1 - 1.30% Coverage

Delivering in a health facility is not a familiar thing to most women; they don’t know the goodness of delivering in a health facility.

Reference 2 - 1.69% Coverage

For sure, they don’t know the goodness of delivering in the health facility. Lying on the bed and spreading the legs is not an issue so long as you can deliver safely at no cost

<Internals¥¥FGD¥¥Women FGD¥¥FGD Biar 14032015_women> - § 2 references coded [0.94% Coverage]

Reference 1 - 0.37% Coverage

If you bring us health education, we will be glad to learn from you.

Reference 2 - 0.57% Coverage

Now, if you can teach us the badness and goodness of the hospital, we shall know more about our health.

<Internals¥¥FGD¥¥Women FGD¥¥FGD Chatom Alor 13032015_women> - § 2 references coded [1.29% Coverage]

Reference 1 - 0.53% Coverage

We don’t give birth in the hospital because we don’t know the importance of hospital delivery.

Reference 2 - 0.77% Coverage

In this community, we don’t know the goodness of the hospital. Before CUAMM came, we were unaware of the benefits of hospital delivery.

<Internals¥¥FGD¥¥Women FGD¥¥FGD Maper 19032015 women> - § 1 reference coded [0.42% Coverage]

Reference 1 - 0.42% Coverage

We don’t know the goodness of delivering in the hospital.

<Internals¥¥FGD¥¥Women FGD¥¥FGD Nhomleng camp2 21032015_women> - § 5 references coded [4.51% Coverage]

Reference 1 - 1.14% Coverage

We have never given birth in the hospital but we deliver here in the cattle camp. We therefore don’t know the goodness or badness of delivering in the hospital.

Reference 2 - 0.91% Coverage

Those that have ever gone to the hospital will know the goodness of the hospital, but for me, I have never gone to the hospital.

Reference 3 - 0.79% Coverage

We are used to delivering in the cattle camp because we don’t know the goodness of delivering in the hospital.

Reference 4 - 1.25% Coverage

It is not because we dislike doctors but it is just that we were not having a hospital before Maper Hospital was established. We really don’t know the goodness of the hospital.

Reference 5 - 0.43% Coverage

All along, we have never known the goodness of the hospital.

<Internals¥¥KIIs¥¥KII CHW Aciek PHCU> - § 1 reference coded [6.70% Coverage]

Reference 1 - 6.70% Coverage

The PHCC was opened in February 2014. For all this time, nobody knew about delivering in a health facility. Some women delivered under a tree, others in the woods. They don’t know the benefit of the health facility.

<Internals¥¥KIIs¥¥KII TBA 5 Malueth> - § 1 reference coded [1.57% Coverage]

Reference 1 - 1.57% Coverage

Women don’t know the goodness of a health facility.

<Internals¥¥KIIs¥¥KII TBA3> - § 2 references coded [13.31% Coverage]

Reference 1 - 7.24% Coverage

Women are not informed that there is a hospital where they can be helped. Myself I deliver in the village, I don’t know the importance of delivering in the hospital.

Reference 2 - 6.06% Coverage

When we go to inform women to come and deliver in the health facility, they ask about the benefit and what they will receive if they come.

<Internals¥¥FGD¥¥Women FGD¥¥FGD Achiek 17032015_women> - § 1 reference coded [0.66% Coverage]

Reference 1 - 0.66% Coverage

Some of us don’t know the goodness of delivering in the hospital.

<Internals¥¥FGD¥¥Women FGD¥¥FGD Biar 14032015_women> - § 4 references coded [3.32% Coverage]

Reference 1 - 0.57% Coverage

Now, if you can teach us the badness and goodness of the hospital, we shall know more about our health.

Reference 2 - 0.98% Coverage

Some people have never known the hospital very well until they are now old. This is because one, the hospital is very far, and two, they don’t know the benefits of the hospital.

Reference 3 - 1.04% Coverage

Some of us have never given birth in the hospital because we don’t know whether the hospital can help or not. We have never understood the reasons why we should give birth in the hospital.

Reference 4 - 0.72% Coverage

For some of us, until we deliver in the hospital first and see whether it is helpful or not, we will continue to deliver at home.

<Internals¥¥FGD¥¥Women FGD¥¥FGD Chatom Alor 13032015_women> - § 2 references coded [1.35% Coverage]

Reference 1 - 0.67% Coverage

We never deliver in health facilities because we don’t know the advantage of delivering there. We just deliver at home.

Reference 2 - 0.68% Coverage

There is nobody among us here who ever delivered in the hospital so we can’t really tell the goodness or badness of it.

<Internals¥¥FGD¥¥Women FGD¥¥FGD Madhol Malueth 12032015_women> - § 1 reference coded [0.37% Coverage]

Reference 1 - 0.37% Coverage

Women usually deliver at home so they don’t know the goodness of the hospital.

<Internals¥¥KIIs¥¥KII CHD M_E> - § 1 reference coded [0.86% Coverage]

Reference 1 - 0.86% Coverage

Some people don’t know the goodness of delivering in the health facility.

<Internals¥¥KIIs¥¥KII CHW Alor> - § 4 references coded [3.93% Coverage]

Reference 1 - 0.94% Coverage

They prefer to deliver at home because they don’t know the goodness of the facility.

Reference 2 - 0.71% Coverage

They don’t know the advantage of delivering in a health facility

Reference 3 - 1.02% Coverage

What I have observed from them is that they don’t know the goodness of this health facility

Reference 4 - 1.26% Coverage

They use traditional medicine and they do not know the goodness of the facility, if they knew they would come.

<Internals¥¥KIIs¥¥KII CHW Maper 4> - § 1 reference coded [2.94% Coverage]

Reference 1 - 2.94% Coverage

Some women don’t understand the benefit of delivering in a health facility.

<Internals¥¥KIIs¥¥KII CHW Meen PHCU> - § 1 reference coded [2.40% Coverage]

Reference 1 - 2.40% Coverage

They don’t know that a health facility can help.

<Internals¥¥KIIs¥¥KII TBA2> - § 1 reference coded [2.94% Coverage]

Reference 1 - 2.94% Coverage

In our community, we don’t know the importance of the hospital.

<Internals¥¥KIIs¥¥KII TBA3> - § 1 reference coded [13.66% Coverage]

Reference 1 - 13.66% Coverage

When we go to inform women to come and deliver in the health facility, they ask about the benefit and what they will receive if they come. They wonder why we bother them to deliver in the health facility. They say that they can just deliver at home because there is nothing to add to it in the health facility.

<Internals¥¥KIIs¥¥KII_Payam admin Meen> - § 3 references coded [9.03% Coverage]

Reference 1 - 2.48% Coverage
Actually this area has been remote, they don’t know the benefit of the hospital.

Reference 2 - 1.92% Coverage
In the past they did not know the goodness of the hospital.

Reference 3 - 4.62% Coverage
The same applies to what I have mentioned before that they don’t know the goodness of the hospital, which is why they don’t deliver in the hospital.

Low risk perception

<Internals¥¥FGD¥¥Men FGD¥¥Achiek_men 17032015_rev> - § 1 reference coded [5.75% Coverage]

Reference 1 - 5.75% Coverage
The reason why some pregnant women in this community don’t visit the hospital is that this community is still held up in traditional way of thinking. We belief that delivery is one of the simplest exercises one can do because even our great grandmothers did it without any problem. Since the beginning of the world, there has been no need for a pregnant woman to deliver in the hospital in this place.

<Internals¥¥FGD¥¥Men FGD¥¥Achiek_men 17032015_rev> - § 1 reference coded [4.31% Coverage]

Reference 1 - 4.31% Coverage
The issue of traditional way of doing things is still in our minds. We think that delivery is the simplest work known to women since creation. There is no need for pregnant women to deliver in the health facility. This is why pregnant women in our community don’t deliver in health facilities.

<Internals¥¥FGD¥¥Men FGD¥¥Meen_men 18032015_rev> - § 2 references coded [3.14% Coverage]

Reference 1 - 1.29% Coverage

Some women don't deliver in the health facility because they are used to delivering at home without any problem.

Reference 2 - 1.84% Coverage

The other reason why a woman may not deliver in a health facility is that if she is healthy, she will feel that she is able to push the child by herself at home.

<Internals¥¥FGD¥¥Women FGD¥¥FGD Biar 14032015_women> - § 1 reference coded [0.81% Coverage]

Reference 1 - 0.81% Coverage

Some women don't experience severe labour pains and so they ignore and say that they can manage to deliver at home because the labour pain is mild.

<Internals¥¥FGD¥¥Women FGD¥¥FGD Nhomleng camp2 21032015_women> - § 1 reference coded [0.57% Coverage]

Reference 1 - 0.57% Coverage

We have been having cattle camps since long time ago and we always deliver there.

<Internals¥¥FGD¥¥Women FGD¥¥FGD Meen village 18032015_women> - § 1 reference coded [0.66% Coverage]

Reference 1 - 0.66% Coverage

Our grandmothers never went to hospitals but they gave birth normally and so we shall follow the way they used to give birth.

<Internals¥¥FGD¥¥Women FGD¥¥FGD Nhomleng camp2 21032015_women> - § 2 references coded [3.80% Coverage]

Reference 2 - 2.33% Coverage

Some women deliver at home because it is our tradition. We are still following the way of our

grandmothers who always delivered at home and not in the hospital. Our grandmothers say that they have ten children whom they delivered at home without anything happening to them. We know that God is our only helper during delivery.

<Internals¥¥KIIs¥¥KII_Payam admin Meen> - § 1 reference coded [7.63% Coverage]

Reference 1 - 7.63% Coverage

In the past people used to say that it is very simple for a woman to deliver, that is what is still in their heads. People still think that delivery is a simple thing and there is no need to go to the hospital. There are no other cultural factors

Medicalization of childbirth

Birth is a natural event

<Internals¥¥FGD¥¥Men FGD¥¥Achiek_men 17032015_rev> - § 1 reference coded [5.75% Coverage]

Reference 1 - 5.75% Coverage

The reason why some pregnant women in this community don’t visit the hospital is that this community is still held up in traditional way of thinking. We belief that delivery is one of the simplest exercises one can do because even our great grandmothers did it without any problem. Since the beginning of the world, there has been no need for a pregnant woman to deliver in the hospital in this place.

<Internals¥¥FGD¥¥Women FGD¥¥FGD Achiek 17032015_women> - § 1 reference coded [1.47% Coverage]

Reference 1 - 1.47% Coverage

For my first three pregnancies, I delivered at home without any problem. For my fourth pregnancy, I got sick and went to the hospital for treatment.

<Internals¥¥FGD¥¥Women FGD¥¥FGD Biar 14032015_women> - § 1 reference coded [2.38% Coverage]

Reference 1 - 2.38% Coverage

It is until you are in labour for almost two days that you will be taken to the hospital to deliver there. The reason you go to the hospital is because of severe labour pain. If you are in severe pain, you will be taken to the hospital to deliver there. With the little pain you experience and then give birth, do you think you need go to the hospital? You go to the hospital when you are almost dying to seek the help of midwives.

<Internals¥¥FGD¥¥Women FGD¥¥FGD Chatom Alor 13032015_women> - § 1 reference coded [3.18% Coverage]

Reference 1 - 3.18% Coverage

Last year when I was pregnant, I fell sick and then went to Maper hospital. I found that the doctor was not there. I was told that he went to Rumbek. I came back home feeling very sick. My sickness increased and within four days I was unable to eat and drink. After five days, I went back to Maper hospital. I got the doctor and I was given medicine which I took, got healed and came back to my house. The doctor asked me to return after a few days and I came home with my medicine and took them. After five days, I delivered at home and my baby was very healthy.

<Internals¥¥FGD¥¥Women FGD¥¥FGD Meen village 18032015_women> - § 1 reference coded [0.62% Coverage]

Reference 1 - 0.62% Coverage

It is only us who know the amount of pain in our bodies, if we cannot manage to deliver at home, we go to the hospital.

<Internals¥¥KIIs¥¥KII_Malueth Chief 1> - § 2 references coded [13.25% Coverage]

Reference 1 - 8.85% Coverage

Our culture is like this: If a woman is pregnant she has to deliver in your presence. If you can only allow your wife to deliver in the health facility if she is sick, otherwise she has to deliver at home. When she is healthy, you cannot allow her to go out.

Reference 2 - 4.41% Coverage

The hospital is good, it can help people. Somebody who is sick and the baby is not well can go to the hospital to deliver there.

Supportive familiar companionship at birth

<Internals¥¥FGD¥¥Women FGD¥¥FGD Maper 19032015 women> - § 1 reference coded [1.14% Coverage]

Reference 1 - 1.14% Coverage

The other reason is that there is nobody to take care of us at the hospital after delivery because we lack transportation means and the hospital is very far.

<Internals¥¥FGD¥¥Women FGD¥¥FGD Meen village 18032015_women> - § 1 reference coded [1.40% Coverage]

Reference 1 - 1.40% Coverage

What I dislike about the hospital is that when you go to deliver in there, you feel like you are in the prison because there is no warm water and porridge given to you, and if there is nobody who accompanied you to take care of you, it will be like you are in the bush.

<Internals¥¥FGD¥¥Women FGD¥¥FGD Nhomleng camp2 21032015_women> - § 1 reference coded [1.97% Coverage]

Reference 1 - 1.97% Coverage

Giving birth at home or in the cattle camp is not bad because there will be many people to help you. For instance, some people will come and hold your shoulders and the other one will come and put you on the lap. If it is in the hospital, there is only the midwife to help you.

<Internals¥¥KIIs¥¥KII CHW Maper 4> - § 1 reference coded [5.17% Coverage]

Reference 1 - 5.17% Coverage

They are also concerned about lack of somebody to take care of them when they come to deliver here. The hospital has no food to eat.

Undesirable birth practice and privacy

<Internals¥¥FGD¥¥Men FGD¥¥Maper_men 19032015_rev> - § 1 reference coded [3.30% Coverage]

Reference 1 - 3.30% Coverage

The delivery position in the hospital whereby a woman is asked by the midwife to lie on bed and spread open her legs discourage women from delivering in health facilities because they are

not comfortable with that delivery position. Traditionally, our women deliver by squatting with knees facing down so that nobody can see their private parts.

<Internals¥¥FGD¥¥Men FGD¥¥Meen_men 18032015_rev> - § 1 reference coded [2.57% Coverage]

Reference 1 - 2.57% Coverage

The other reason is the delivery position used in the health facility. In the health facility, the midwife asks the woman to sleep facing up and to spread wide her legs. Women in this village are not used to that position.

<Internals¥¥FGD¥¥Women FGD¥¥FGD Madhol Malueth 12032015_women> - § 1 reference coded [0.69% Coverage]

Reference 1 - 0.69% Coverage

The other reason why we don’t like the hospital is that when you are in labour they position you facing up with legs open; we don’t like that position .

<Internals¥¥FGD¥¥Women FGD¥¥FGD Meen village 18032015_women> - § 1 reference coded [1.78% Coverage]

Reference 1 - 1.78% Coverage

We don’t like the delivery position in the hospital whereby a woman lays down and opens her legs wide. It is not a good position of giving birth according to our imagination. But in the village or at home, we just squat with legs slightly open and somebody pushes the mother down on the shoulders while the TBA is in front waiting for the baby.

<Internals¥¥FGD¥¥Women FGD¥¥FGD Nhomleng camp2 21032015_women> - § 3 references coded [4.00% Coverage]

Reference 1 - 1.06% Coverage

In the hospital, the baby may not get fresh air because it will be covered immediately after birth, but in a cattle camp, the baby will get fresh air.

Reference 2 - 1.57% Coverage

While in the hospital during delivery, a pregnant woman is laid down facing up with legs apart. Then sometimes, all her clothes are removed and she is left naked. For this reason, I don’t like

delivering in the hospital.

Reference 3 - 1.37% Coverage

We also don't like how the placenta is handled for example the placenta is thrown in deep a pit. Because of the throwing of the placenta in a pit, I don't like to deliver in the hospital.

<Internals¥¥KIIs¥¥KII CHW Maper 2> - § 1 reference coded [5.91% Coverage]

Reference 1 - 5.91% Coverage

The positioning of the woman on the delivery bed is a problem for them. They prefer squatting. That is the main thing they say.

<Internals¥¥KIIs¥¥KII TBA1> - § 1 reference coded [6.73% Coverage]

Reference 1 - 6.73% Coverage

Women don't like vaginal examination. In the hospital, they put on gloves to do vaginal examination to measure whether the woman will deliver well or not, and women do not like this.

<Internals¥¥FGD¥¥Women FGD¥¥FGD Madhol Malueth 12032015_women> - § 4 references coded [5.99% Coverage]

Reference 1 - 1.49% Coverage

Traditionally, women are the ones who help us. They hold you at the shoulders as you squat during delivery and the TBA will be waiting with open hands below to receive the baby. It is yourself who will be responsible for pushing the child, not somebody else. Even if it is three days, people will be waiting for you to deliver.

<Internals¥¥KIIs¥¥KII CHD M_E> - § 2 references coded [4.23% Coverage]

Reference 1 - 1.04% Coverage

Some women are also shy. If they see that the hospital I full, they will refuse to come.

Reference 2 - 3.18% Coverage

Some women say that when you go to a health facility for delivery, that is not good because delivering at home is good for tradition. At home there is privacy but in the health facility there is no privacy and women are asked to deliver in the position they are not used to.

<Internals¥¥KIIs¥¥KII CHW Maper 4> - § 2 references coded [13.01% Coverage]

Reference 1 - 8.58% Coverage

The clinic is very small and cannot accommodate many people. Some people don't want to come because the health facility is too crowded with many people mixed up. They say that they don't want to be seen by many people.

Reference 2 - 4.43% Coverage

They don't want their nakedness to be seen by health personnel, they are comfortable to be seen only by the TBAs.

Fear of caesarean section

<Internals¥¥FGD¥¥Men FGD¥¥Maper_men 19032015_rev> - § 1 reference coded [1.48% Coverage]

Reference 1 - 1.48% Coverage

Another reason is that they are afraid of caesarean section. They are worried about not being able to continue producing children after caesarean section.

<Internals¥¥FGD¥¥Women FGD¥¥FGD Madhol Malueth 12032015_women> - § 1 reference coded [1.50% Coverage]

Reference 1 - 1.50% Coverage

In the hospital, there are some things we don't like. For example, if you are in labour for three days and you are not delivering easily, the doctor might decide to operate on you. After you have been operated and they have brought out the child, they will stitch you. This is the reason I don't like delivering in the hospital.

<Internals¥¥FGD¥¥Women FGD¥¥FGD Maper 19032015 women> - § 1 reference coded [0.38% Coverage]

Reference 1 - 0.38% Coverage

Some of us fear cesarean section in the hospital.

<Internals¥¥FGD¥¥Women FGD¥¥FGD Nhomleng camp2 21032015_women> - § 1 reference coded [0.79% Coverage]

Reference 1 - 0.79% Coverage

The reasons we don’t deliver in the hospital is that pregnant women may be operated when in severe labour pain.

Perceptions about quality of care

Health facility infrastructure and commodities

<Internals¥¥FGD¥¥Men FGD¥¥Ror bar_men 16032015_rev> - § 4 references coded [5.38% Coverage]

Reference 1 - 1.84% Coverage

When we were in the bush, we were suffering a lot without health facilities but now we have some PHCUs in our areas. However, at the moment, when you take your wife to the hospital *(phcu)*, you find that there are no drugs.

Reference 2 - 1.13% Coverage

When a woman goes to the health facility, she gets no medicine for her to survive.

Now we have a health facility but we lack medicine.

Reference 3 - 1.82% Coverage

The only problem with our health facility is lack of some instruments and the laboratory for testing diseases to be treated. We request your organisation to provide us with different types of instruments and a laboratory.

Reference 4 - 0.58% Coverage

My wife was sick and I took her to the health unit but found no doctor.

<Internals¥¥FGD¥¥Women FGD¥¥FGD Achiek 17032015_women> - § 3 references coded [3.33% Coverage]

Reference 1 - 1.00% Coverage

What we need now is for the hospital to be expanded because we don’t have drugs for various diseases.

Reference 2 - 1.04% Coverage

The hospital is very far from us and there are no means of transportation to take us there during labour.

Reference 3 - 1.28% Coverage

In the hospital, one midwife is not enough to attend to delivering mothers at the same time. Other midwives are not well trained.

<Internals¥¥FGD¥¥Women FGD¥¥FGD Meen village 18032015_women> - § 1 reference coded [0.67% Coverage]

Reference 1 - 0.67% Coverage

Our hospital in Meen here is not like the one in Maper; a woman cannot deliver here because there is no good room for maternity.

<Internals¥¥FGD¥¥Women FGD¥¥FGD Nhomleng campe1 21032015_women> - § 1 reference coded [1.13% Coverage]

Reference 1 - 1.13% Coverage

Sometimes even if we go to the hospital, we find that some drugs are not there and then we are told to buy, yet we don’t have money.

<Internals¥¥KIIs¥¥KII CHD M_E> - § 2 references coded [3.46% Coverage]

Reference 1 - 1.27% Coverage

First all we don’t have enough facilities where women can deliver. Our PHCC is new and is the only one here.

Reference 2 - 2.19% Coverage

The other problem is lack of a spacious building; this building here is small and cannot accommodate all the women who may want to deliver in a health facility, because it is only one room.

<Internals¥¥KIIs¥¥KII CHW Alor> - § 4 references coded [4.39% Coverage]

Reference 1 - 2.01% Coverage

They do attend antenatal care, but deliver at home. We have nothing to give like basins, mosquito net during birth. There is no room for delivery here. These are the major issues.

Reference 2 - 0.87% Coverage

Even though a woman may want to deliver here, lack of space can affect her.

Reference 3 - 0.39% Coverage

There is no equipment for delivery.

Reference 4 - 1.12% Coverage

There are drugs which are not here. I don’t have a place to admit mothers who want to deliver here.

<Internals¥¥KIIs¥¥KII CHW Maper 1> - § 1 reference coded [8.86% Coverage]

Reference 1 - 8.86% Coverage

We should get other means of transport such as motorcycle ambulances, and the maternity should be built. This maternity is now enough. Now all patients including women are being admitted in one ward.

<Internals¥¥KIIs¥¥KII CHW Maper 2> - § 1 reference coded [7.51% Coverage]

Reference 1 - 7.51% Coverage

The PHCC is good but there is need for the PHCUs to also have delivery couches and the incentives provided there (mama kits). All facilities should also have EPI.

<Internals¥¥KIIs¥¥KII CHW Meen PHCU> - § 1 reference coded [7.89% Coverage]

Reference 1 - 7.89% Coverage

If the pregnant woman comes and she has a big problem, we refer her to Maper. We don’t have a room, materials for screening and pieces of cloth for the baby etc**.**

<Internals¥¥KIIs¥¥KII TBA Meen> - § 1 reference coded [11.01% Coverage]

Reference 1 - 11.01% Coverage

The only reason why women deliver at home is because there is no an accommodative facility where they can deliver. Also the health facility has no delivery services/equipment.

<Internals¥¥KIIs¥¥KII TBA3> - § 2 references coded [15.10% Coverage]

Reference 1 - 8.73% Coverage

Another issue is that there is not enough space at the health facility; it is very small. We need a spacious delivery room so that when a woman is in labour, she can be comfortable in the labour room.

Reference 2 - 6.37% Coverage

We lack many things such as delivery couches. When a mother comes, we have nothing to do.

We also don't have medicines and vaccines for children.

<Internals¥¥KIIs¥¥KII_Malueth Chief 2> - § 3 references coded [26.59% Coverage]

Reference 1 - 19.24% Coverage

Whenever a woman is pregnant she must go to the hospital. When it comes to the time of delivery, we lack good room where a woman can deliver. What is here in Malueth? The main medicines for certain diseases are not there. Those medicines are there in Maper. Even if a pregnant woman is about to deliver, we don't have a good room where the woman can deliver. We have to take that woman to the hospital.

Reference 2 - 4.30% Coverage

The reason why they deliver at home is that there is no maternity at our PHCU of Malueth.

Reference 3 - 3.05% Coverage

We are lack medicine in our place. Our PHCU is not organised.

Neglect and lack of communication

<Internals¥¥FGD¥¥Women FGD¥¥FGD Meen village 18032015_women> - § 2 references coded [3.22% Coverage]

Reference 1 - 1.40% Coverage

What I dislike about the hospital is that when you go to deliver in there, you feel like you are in the prison because there is no warm water and porridge given to you, and if there is nobody who accompanied you to take care of you, it will be like you are in the bush.

Reference 2 - 1.82% Coverage

Like me when I went to deliver in the hospital, I was just put in a car to take me to the hospital and after I had delivered, the midwife and the doctors left me alone in the maternity and went away. I slept there without water for showering and food. That was not good. When somebody is not there to take care of you, it is better to deliver at home.
